# Bioelectronic Medicine: a multidisciplinary roadmap from biophysics to precision therapies

**DOI:** 10.3389/fnint.2024.1321872

**Published:** 2024-02-19

**Authors:** María Alejandra González-González, Silvia V. Conde, Ramon Latorre, Stéphanie C. Thébault, Marta Pratelli, Nicholas C. Spitzer, Alexei Verkhratsky, Marie-Ève Tremblay, Cuneyt G. Akcora, Ana G. Hernández-Reynoso, Melanie Ecker, Jayme Coates, Kathleen L. Vincent, Brandy Ma

**Affiliations:** ^1^Jan and Dan Duncan Neurological Research Institute, Texas Children’s Hospital, Houston, TX, United States; ^2^Department of Pediatric Neurology, Baylor College of Medicine, Houston, TX, United States; ^3^iNOVA4Health, NOVA Medical School, Faculdade de Ciências Médicas, NOVA University, Lisbon, Portugal; ^4^Centro Interdisciplinario de Neurociencia de Valparaíso, Facultad de Ciencias, Universidad de Valparaíso, Valparaíso, Chile; ^5^Laboratorio de Investigación Traslacional en salud visual (D-13), Instituto de Neurobiología, Universidad Nacional Autónoma de México (UNAM), Querétaro, Mexico; ^6^Neurobiology Department, Kavli Institute for Brain and Mind, UC San Diego, La Jolla, CA, United States; ^7^Faculty of Biology, Medicine and Health, The University of Manchester, Manchester, United Kingdom; ^8^Achucarro Centre for Neuroscience, IKERBASQUE, Basque Foundation for Science, Bilbao, Spain; ^9^Department of Forensic Analytical Toxicology, School of Forensic Medicine, China Medical University, Shenyang, China; ^10^International Collaborative Center on Big Science Plan for Purinergic Signaling, Chengdu University of Traditional Chinese Medicine, Chengdu, China; ^11^Department of Stem Cell Biology, State Research Institute Centre for Innovative Medicine, Vilnius, Lithuania; ^12^Division of Medical Sciences, University of Victoria, Victoria, BC, Canada; ^13^Department of Neurology and Neurosurgery, McGill University, Montreal, QC, Canada; ^14^Department of Molecular Medicine, Université Laval, Québec City, QC, Canada; ^15^Department of Biochemistry and Molecular Biology, The University of British Columbia, Vancouver, BC, Canada; ^16^Department of Computer Science, University of Central Florida, Orlando, FL, United States; ^17^Department of Bioengineering, The University of Texas at Dallas, Richardson, TX, United States; ^18^Department of Biomedical Engineering, University of North Texas, Denton, TX, United States; ^19^The Luxi Group, New Hartford, CT, United States; ^20^Department of Obstetrics and Gynecology, University of Texas Medical Branch, Galveston, TX, United States; ^21^Stanley H. Appel Department of Neurology, Houston Methodist Hospital, Houston, TX, United States

**Keywords:** bioelectronic medicine, neuromodulation, channel biophysics, glia, neuronal plasticity, high throughput data, biocompatible materials, medical devices

## Abstract

Bioelectronic Medicine stands as an emerging field that rapidly evolves and offers distinctive clinical benefits, alongside unique challenges. It consists of the modulation of the nervous system by precise delivery of electrical current for the treatment of clinical conditions, such as post-stroke movement recovery or drug-resistant disorders. The unquestionable clinical impact of Bioelectronic Medicine is underscored by the successful translation to humans in the last decades, and the long list of preclinical studies. Given the emergency of accelerating the progress in new neuromodulation treatments (i.e., drug-resistant hypertension, autoimmune and degenerative diseases), collaboration between multiple fields is imperative. This work intends to foster multidisciplinary work and bring together different fields to provide the fundamental basis underlying Bioelectronic Medicine. In this review we will go from the biophysics of the cell membrane, which we consider the inner core of neuromodulation, to patient care. We will discuss the recently discovered mechanism of neurotransmission switching and how it will impact neuromodulation design, and we will provide an update on neuronal and glial basis in health and disease. The advances in biomedical technology have facilitated the collection of large amounts of data, thereby introducing new challenges in data analysis. We will discuss the current approaches and challenges in high throughput data analysis, encompassing big data, networks, artificial intelligence, and internet of things. Emphasis will be placed on understanding the electrochemical properties of neural interfaces, along with the integration of biocompatible and reliable materials and compliance with biomedical regulations for translational applications. Preclinical validation is foundational to the translational process, and we will discuss the critical aspects of such animal studies. Finally, we will focus on the patient point-of-care and challenges in neuromodulation as the ultimate goal of bioelectronic medicine. This review is a call to scientists from different fields to work together with a common endeavor: accelerate the decoding and modulation of the nervous system in a new era of therapeutic possibilities.

## 1 Bioelectronic medicine (*M. A. González-González, S. V. Conde*)

The concept of Neuromodulation is defined by the International Neuromodulation Society (INS) as the use of advanced medical device technology to enhance or suppress the activity of the nervous system for the treatment of disease. This concept has evolved to “Bioelectronic Medicine” when using electrical current delivered to the nervous tissue (central or peripheral) to achieve targeted therapeutic benefits (Birmingham et al., 2014; Pavlov and Tracey, 2019, 2022; Cho et al., 2020). While this is a recent concept, early reports of the use of electricity as a therapy date back to thousands of years BC. The Ancient Egyptians and later the Greeks and Romans identified that electrical torpedo fishes generate electrical shocks that provide pain relief (Kane and Taub, 1975; Dolhem, 2008; Heidland et al., 2013). In the 18^th^ and 19^th^ centuries, these natural processes of electricity generation were replaced by man-made electrical devices. In the 18^th^ century, Galvani’s momentum marked an era of discovery with his observations on frog’s leg contractions when applying electrical current over nerves or tissue (Kane and Taub, 1975; Loeb, 2014). Then, Benjamin Franklin led the generation of static electrical currents with his electrostatic machine and demonstrated the therapeutic effects of electroconvulsive shock therapy. This era was documented as Franklinism (Beaudreau and Finger, 2006; Finger, 2006; Finger and Zaromb, 2006).

The 19th century was the “golden age” of electrotherapy. In 1831, Michael Faraday fabricated the first electric generator apparatus, that produced intermittent current. Then Wilhelm Holtz developed the Holtz machine, a generator of static electrical current, which was implemented in medical practice to relieve pain and migraine (Al-Khalili, 2015). At the end of the 19th century, electricity was used for countless dental, neurological, psychiatric and gynecological conditions. However, at the beginning of the 20th century, electrotherapy lacking a scientific basis lost credibility. Furthermore, the development of effective analgesic drugs decreased the interest in electricity (Fodstad and Hariz, 2007; Dolhem, 2008). In the second half of the 20th century, electrotherapy underwent a revival. Based on animal experiments and clinical investigations, its neurophysiological mechanisms were elucidated in more detail. The pain-relieving action of electricity was explained in particular by two main mechanisms: first, segmental inhibition of pain signals to the brain in the dorsal horn of the spinal cord and second, activation of the descending inhibitory pathway with enhanced release of endogenous opioids and other neurochemical compounds (e.g., serotonin, noradrenaline, gamma aminobutyric acid, acetylcholine and adenosine) (Dolhem, 2008; Vallejo et al., 2017; Chakravarthy et al., 2018).

**Figure 1:**
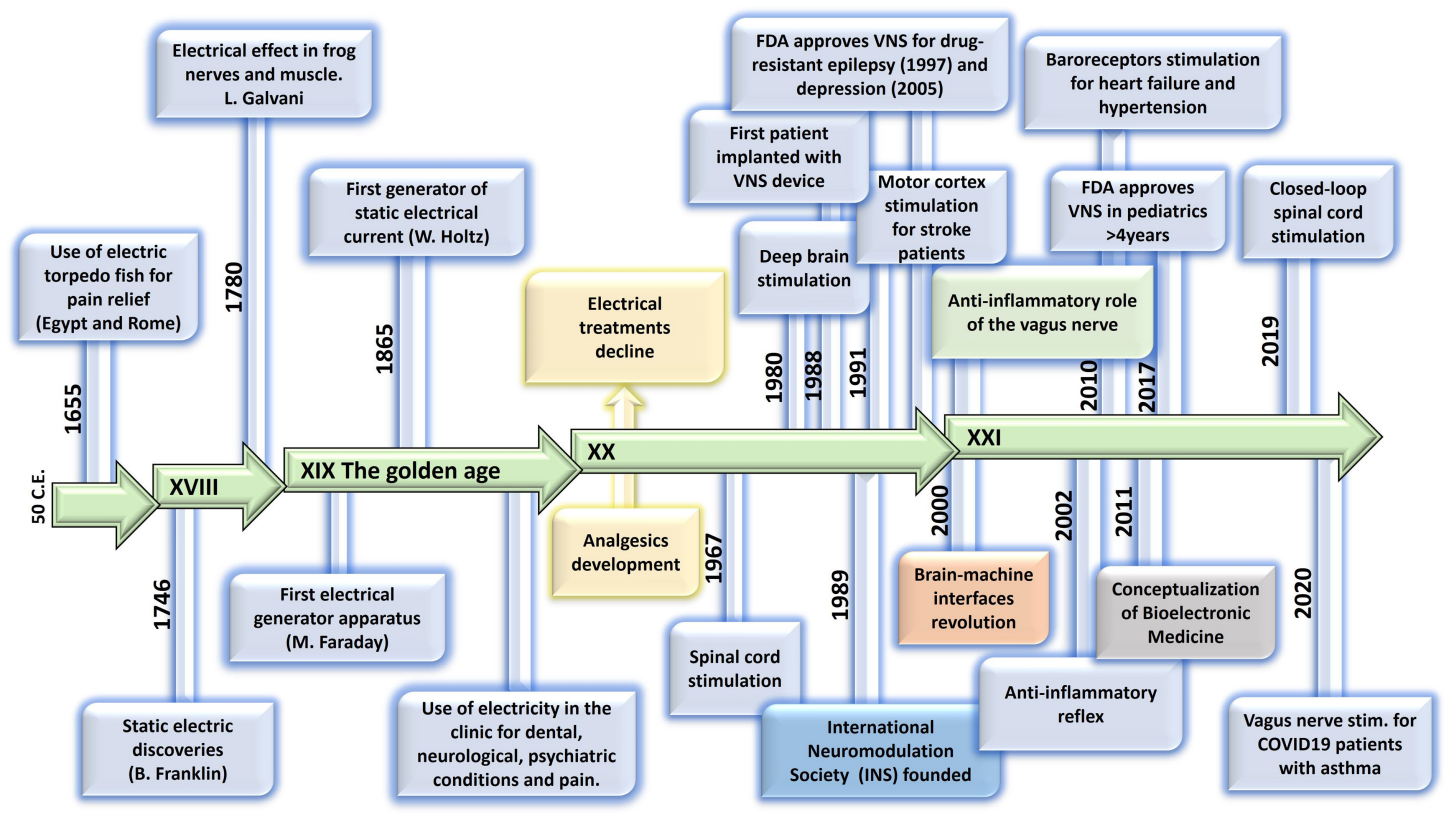
Evolution of electrical therapies. Timeline highlighting the main historic events and evolution of the use of electricity for neuromodulation therapies. XIX century is recognized as the golden age due to impactful discoveries in electricity, including the first electrical generator apparatus (by M. Faraday) and the first generator of static electrical current (by W. Holtz). At XX century, the use of electrical therapies declined and lost credibility due to the lack of scientific basis, and to the development of the pharmaceutic industry (incorporation of analgesics). A new era begins at XXI century with the brain-machine interfaces and the discovery of the neuroinflammatory reflex.

The notion of using the stimulation of large nerve fibers in the dorsal columns of the spinal cord for addressing chronic pain emerged during the 1960s (Shealy et al., 1967). This idea reached its pinnacle in 1968 with the development of the first commercial implantable stimulator designed for managing chronic pain and laid the foundation for modern neuromodulation techniques (Gildenberg, 2006). Another milestone in the neuromodulation field was the development of deep brain stimulation (DBS). While first used for the study of the physical manifestations of human emotion (Oliveria, 2018), it was when used for stimulation of the basal ganglia to improve symptoms of Parkinson’s disease that revolutionized the field evolving afterwards into a versatile therapy with applications in various neurological and psychiatric conditions (Frey et al., 2022). It was in the late 1980s that modern DBS techniques gained attention, with the advent of precise electrode placement and sophisticated neuroimaging methods. By delivering controlled electrical impulses, DBS modulates aberrant neural activity, effectively alleviating symptoms in conditions such as Parkinson’s disease, essential tremor, dystonia, epilepsy, obsessive-compulsive disorder, and even depression and schizophrenia. Ongoing advancements in electrode design, longer-lasting batteries, programming algorithms for closed-loop approaches, and the understanding of neural circuitry help refine and expand the therapeutic possibilities of DBS, offering new avenues of hope for individuals grappling with complex neurological and psychiatric disorders (Frey et al., 2022), and highlighting the necessity of interdisciplinary efforts. In 1989 the INS was funded in order to promote the dialog between clinicians, scientists and engineers (INS, 2022), and since then, national neuromodulation society chapters have been established in more than twenty countries around the globe, who meet in annual global and regional meetings to discuss advances in the field. Figure 1 summarizes the main events in the history of Bioelectronic Medicine.

An important advancement in the bioelectronic field was the implementation of vagus nerve stimulation (VNS). The vagus nerve is the main autonomic nerve communicating the brain with peripheral organs (Baker and Lui, 2019). The approval of VNS for drug-resistant epilepsy in 1997 and subsequently for treatment-resistant depression in 2005 stands out as important milestones within the bioelectronics field (Johnson and Wilson, 2018; Austelle et al., 2022). In the USA, the green light from regulatory authorities FDA for VNS therapy in these conditions has validated the safety and efficacy of bioelectronic interventions. However, the noncoverage determination in 2006 by the Centers for Medicare and Medicaid Services (CMS) that the VNS for depression remained unproven has limited its use (Aaronson and Conway, 2018). More recently, the discovery of transcutaneous auricular vagus nerve stimulation (tVNS) as a viable treatment option for epilepsy and depression carries immense importance for the treatment of these conditions. This innovation not only expands the range of available treatments but also opens the doors for a wider patient population to access therapy and eliminates the need for invasive procedures, reducing potential risks and improving patient comfort and compliance (Verma et al., 2021). It is worth mentioning the limitations of this approach, a customized treatment is required, and often side effects arise due to the off-target stimulation −undesired nerve fibers are recruited (Kim et al., 2022). Section 12 will provide more details on this.

**Figure 2:**
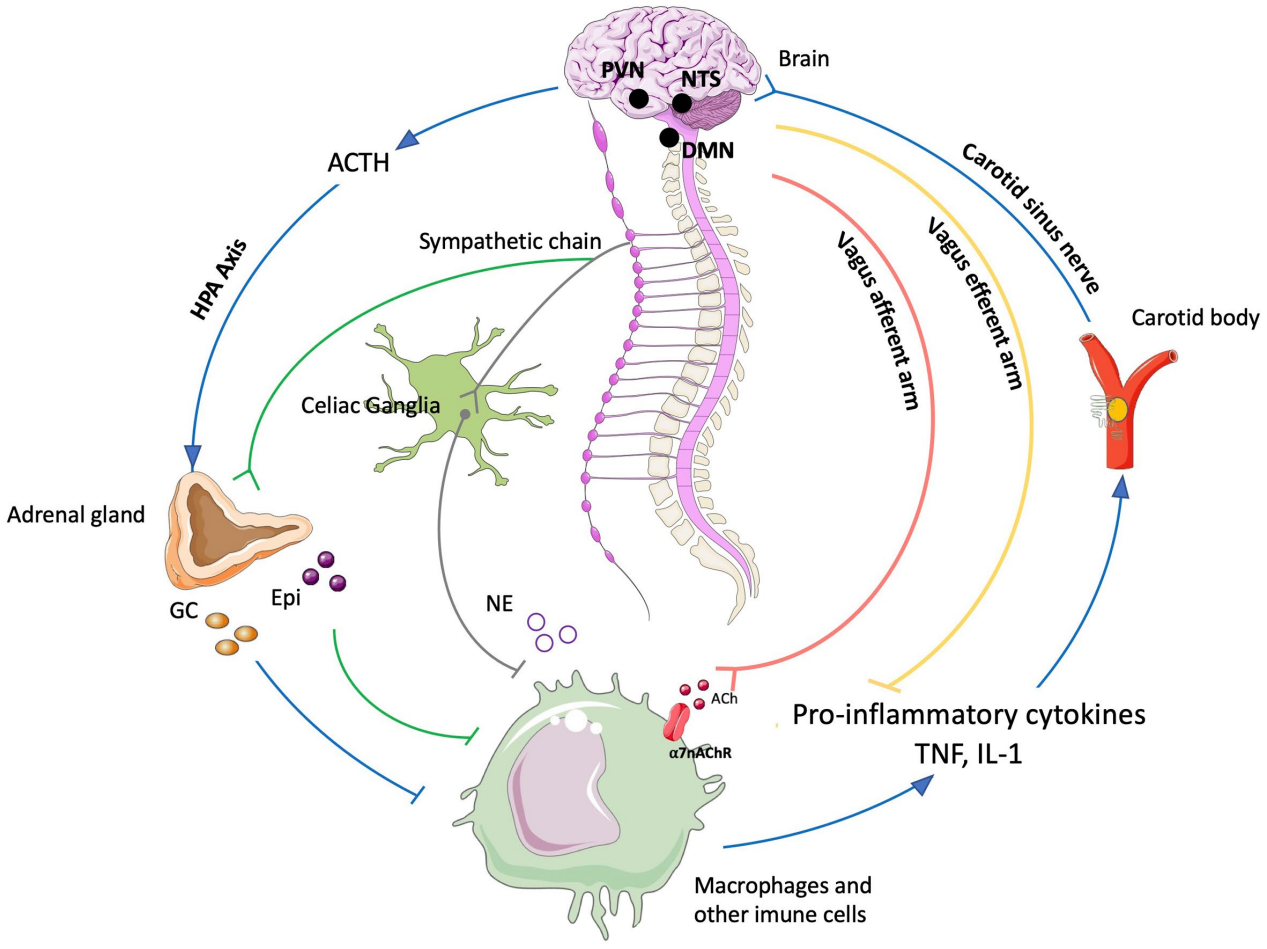
The neuro-immune reflex. The activation of the hypothalamic-pituitary-adrenal (HPA) axis is prompted by various stimuli, initiating a sequence that involves the paraventricular nucleus (PVN) of the hypothalamus. This, in turn, triggers the release of cortisol releasing hormone (CRH) into the anterior pituitary. Consequently, CRH prompts the secretion of adrenocorticotrophic hormone (ACTH) into the bloodstream. ACTH stimulates the adrenal cortex to produce glucocorticoids (GC), which are potent anti-inflammatory molecules. These effects are mediated by the glucocorticoid receptor (GR), a nuclear receptor present in almost all cells, particularly innate immune cells. The vagal anti-inflammatory reflex is characterized by peripheral vagal afferent nerves sensing inflammation and conveying this information to the central nervous system (CNS). The nucleus tractus solitary (NTS) and dorsal motor nucleus (DMN) establish connections to communicate and activate efferent vagus nerve fibers. This activation leads to the release of acetylcholine (ACh), which diminishes cytokine production and inflammation. A key mechanism in this process is the binding of ACh to the nicotinic α7nAChR receptor. Furthermore, cytokines can directly activate this specific brain region through humoral control to counteract inflammation. Sympathetic fibers originating in the spinal cord directly innervate visceral organs and immune cells. Upon binding to their receptors, norepinephrine (NE) released from sympathetic fibers and at the celiac ganglia, as well as epinephrine (Epi) from adrenal chromaffin cells, collectively inhibit inflammation and the release of inflammatory cytokines. Additionally, inflammation resolution is facilitated by the actions of the carotid body. Inflammatory mediators released by immune cells trigger the activation of the carotid body, which subsequently stimulates the carotid sinus nerve. This nerve projects into the NTS, contributing to the overall process of inflammation resolution. Created with BioRender.com.

The last decades have been fruitful in the identification of new targets for the treatment of a variety of pathologies using Bioelectronic Medicine, particularly on the peripheral nervous system (PNS). One example is the neuro-immune reflex and its relevance in chronic inflammatory and immune diseases (Eberhardson et al., 2020; Pavlov et al., 2020). The mechanisms behind the neuro-immune reflex have been under debate, but the clinical output has marked a significant turning point in the Bioelectronic Medicine field (Tracey, 2002; Conde et al., 2020; Hu et al., 2020; Pavlov et al., 2020; Gonzalez-Gonzalez et al., 2021; Figure 2). Since pioneer studies in 2000, showed the decrease in inflammation followed by VNS in rats (Borovikova et al., 2000), multiple studies in animal models were performed showing that VNS decreases inflammation and mitigates disease in acute inflammatory conditions such as endotoxemia, sepsis, hemorrhagic shock, postoperative ileus, and kidney ischemia–reperfusion injury [for a review see Kelly et al., 2022] and in chronic inflammatory conditions such as rheumatoid arthritis, inflammatory bowel disease (IBD) and asthma (Levine et al., 2014; Yuan and Silberstein, 2016; Wheless et al., 2018; Austelle et al., 2022). The mechanisms have been under debate and accepted statements include the regulation of cytokine production through the nicotinic acetylcholine receptor subunit α7 (α7nAchR) in the spleen, termed “the cholinergic anti-inflammatory pathway” (Wang et al., 2003; Gallowitsch-Puerta and Pavlov, 2007; Parrish et al., 2008), through second-order vagal projections (indirect) (Ackerman et al., 1987; Berthoud and Powley, 1993) and first-order (direct pathway) vagus nerve-spleen (Gonzalez-Gonzalez et al., 2021). Another important landmark for the bioelectronic targeting of the vagal anti-inflammatory reflex has been the clinical testing of VNS devices for patients with inflammatory and autoimmune disorders, such as rheumatoid arthritis and IBD, with decreased inflammation and positive clinical outcomes (Koopman et al., 2016; Genovese et al., 2020).

Exploring the neuro-immune reflex, beyond the vagus nerve, the electrical modulation of carotid body chemoreceptors also modifies the inflammatory response during sepsis. Previous studies showed in conscious rats, that the electrical stimulation of the carotid sinus nerve, the sensitive nerve of the carotid body, attenuates the response to bacterial lipopolysaccharide (LPS) by decreasing tumor necrosis factor (TNF), interleukin (IL) 1β (IL-1β) and IL-6 levels, and increasing the levels of IL-10. These effects were mediated by both sympathetic and parasympathetic pathways, and therefore consistent with the activation of both afferent and efferent pathways of the anti-inflammatory reflex (Santos-Almeida et al., 2017). In addition, it was shown that the electrostimulation of the carotid sinus nerve in mice attenuates inflammation via glucocorticoid receptors on myeloid immune cells (Falvey et al., 2020). In 2020, a handheld VNS device was authorized by the U.S. FDA as an emergency for asthmatics with COVID-19 experiencing difficulty breathing (ClinicalTrials.gov; identifier: NCT04368156), and the consecutive randomized clinical trial demonstrated potential to mitigate some of the symptoms by reduction of inflammatory markers (Tornero et al., 2022).

The notion of joining forces to accelerate discoveries for Bioelectronic Medicine, led in 2022 to celebrate the 5^th^ Bioelectronic Medicine Summit in NY city, where an update of recent advances was discussed (Ashe et al., 2023). These include the use of targeted ultrasound stimulation, the implementation of solid-state batteries for the miniaturization of medical devices, advances in closed-loop neuromodulation and multi-contact implantable neuronal interfaces. While we are in a revolutionary technological era, multidisciplinary dialog is imperative, including but not limited to clinicians, physiologists, data analysts, and material/electronic engineers. We must keep in mind that neuromodulation goes far beyond VNS, DBS, or spinal cord stimulation. Countless opportunities arise from the discovery of new homeostatic neural mechanisms, and more significantly, from innovative mechanisms and points of intervention. Furthermore, many of the previously identified targets, such as nerves and neuronal regions, have the potential to serve as neuromodulation targets for multiple diseases, emphasizing the importance of acquiring further insights into comprehensive neuroanatomy, neurophysiology, and biophysics needed for the intelligent design of device hardware computational models and stimulation protocols.

The present work provides and discusses core concepts required to understand the Bioelectronic Medicine field. Experts will go from the inner core of neuromodulation to the patient point of care, passing through the current neuromodulation approaches and neural interfaces available and their applications, which altogether stresses the necessity of multidisciplinary dialog to advance this field.

## 2 Biophysics of the neuronal membrane: the inner core of neuromodulation (*R. Latorre*)

Neuronal membrane excitability is a fundamental property underlying function in the nervous system and it depends on a cascade of events followed by a stimulus. In electrical therapy, an external stimulus modifies the neuronal dynamics, and it is necessary to understand the underlying cellular events in order to develop high-precision treatments. This section reviews fundamental concepts of biophysics related to the neuronal membrane, focusing on the main properties of voltage-gated (dependent) channels involved in the adequate functioning of the nervous system.

### 2.1 How voltage-dependent channels are activated by voltage

Since the advent of the patch-clamp technique, followed by ion channel cloning, and ending with the flourishing of structural methods (such as X-ray and cryo-electron microscopy), we have witnessed how the ion channel field has advanced with amazing speed. It would be impossible to examine in detail all the gating and structural properties of the myriad voltage-dependent channels present in the nervous system. Therefore, a bird’s-eye view will be provided to the main biophysical properties.

#### 2.1.1 Membrane equivalent circuit

**Figure 3:**
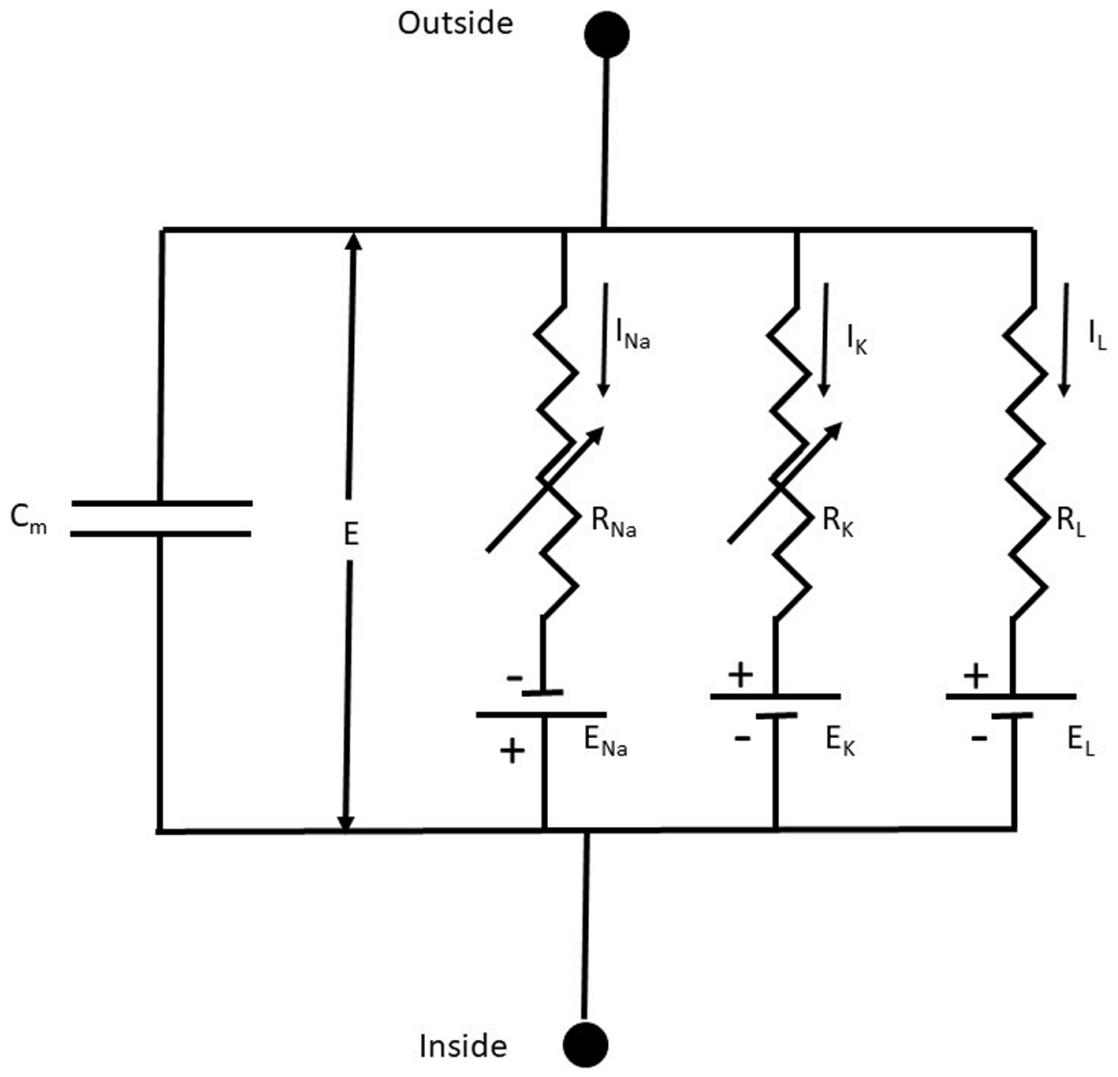
The equivalent membrane electrical circuit. *C_M_* is the membrane capacitor; E is the membrane potential; *E_Na_, E_K_*, and *E_L_* are the equilibrium potentials for Na^+^, K^+^, and L (leakage), respectively. R_Na_, R_K_, and R_L_, are the membrane resistances for Na^+^, K^+^, and L, respectively. Notice that *R_Na_, R_K_* are variable membrane resistances. The currents *I_X_ = G_X_(E – E_X_),* where X = Na^+^, K^+^ or L and the conductance G_X_ = 1/R_X_. From Hodgkin and Huxley (1952), Figure 1.

An excellent starting point to discuss the membrane’s electrical properties is the 1952 paper of Hodgkin and Huxley (HH) (Hodgkin and Huxley, 1952), who summarized their previous findings by representing the Na^+^, K^+^, and leak (L) conductance in an equivalent membrane electrical circuit (Figure 3).

The membrane conductances for Na^+^ and K^+^ (G_Na_ and G_K_, respectively), are variable and are a function of the voltage-dependent membrane potential and time. The leakage conductance (GL) is constant. Thus, according to the present electrophysiological convention, we have a negative inward (IN) current when the membrane potential I < E_Na_, which becomes positive when E > E_Na_. Thus, in this seminal work, for the first time is used the concept of voltage-dependent conductances. The probability of finding the sodium or the potassium conductance operative is a function of voltage. G_Na_ and G_K_ increase with membrane depolarizations (i.e., membrane potentials larger than the resting membrane potential).

Hodgkin and Katz (1949), following the lead of Goldman (1943), obtained an expression for the membrane potential (E_m_) that takes into account internal and external concentrations of K^+^, Na^+^, and Cl^-^ and the permeability coefficients (P) for the different ions. This equation, known as the Goldman-Hodgkin-Katz or constant-field equation, considers the non-equilibrium conditions of the cell system and is still used when describing the ion selectivity of a given conductance system (Alvarez and Latorre, 2017).


(1) $$E_{m}=-\frac{RT}{F}\ln\frac{P_{K}K_{\mathrm{in}}+P_{Na}Na_{\mathrm{in}}+P_{Cl}Cl_{\mathrm{out}}}{P_{K}K_{\mathrm{out}}+P_{Na}Na_{\mathrm{out}}+P_{Cl}Cl_{\mathrm{in}}}$$

The use of Equation (1) allowed Hodgkin and Katz to determine the relative permeabilities of K^+^ and Na^+^ during rest and at the peak of the action potential. At rest, P_K_/P_Na_ = 25, and at the peak of the action potential, P_K_/P_Na_ = 0.05, implying that during an action potential P_Na_ increases 500 times compared with that at rest.

#### 2.1.2 Voltage-dependent conductances

Voltage-dependent channels are constituted by proteic subunits embedded in the cellular membrane, and every subunit is formed by six transmembrane segments (S1-6). These subunits form a central pore that allows the flow of ions, and its opening is triggered by stoichiometric modifications activated by changes in charges. The S4 transmembrane segment in voltage-dependent channels contains the gating charges. HH were fully aware that in order to have a voltage-dependent conductance, charges or dipoles contained in the plasma membrane should interact with the electric field (Hodgkin and Huxley, 1952). The movement of these charged particles (voltage sensors) should induce a current (gating currents), and they were revealed by Armstrong and Bezanilla (1973). The cloning of the voltage-dependent Na^+^ (Na_v_) and the Shaker K^+^ channel (Noda et al., 1984; Papazian et al., 1987; Timpe et al., 1988) gave the first hints about the molecular nature of the voltage sensor. Na_v_ channels are formed by a protein containing four domains, each containing six transmembrane segments (S1-S6). Voltage-dependent K^+^ (K_v_) channels are tetramers; each subunit has six transmembrane segments. Notably, in Na_v_ and K_v_ channels, in the fourth S4 segment, every third residue is a positively charged amino acid residue (arginine or lysine) separated by two hydrophobic residues. Using the limiting slope method (Almers, 1978; Sigg and Bezanilla, 1997) and the total gating charge (Q) divided by the number of channels (N) contained in the same membrane area (Q/N method), it was possible to obtain the number of gating charges per channel. Twelve electronic charges (e0) are needed to be displaced in the electric field to open Na_v_ channels from skeletal muscle (Hirschberg et al., 1995), and 12.3 e0 were obtained in the case of the Shaker K^+^ channel (Schoppa et al., 1992). Using these methodologies to determine the numbers of gating charges per channel and replacing the charged amino acids contained in S4 for the neutral glutamine or asparagine, Aggarwal and MacKinnon (1996) and Seoh et al. (1996) demonstrated that the first four charges from the N-terminal of S4 contribute to the Shaker K^+^ channel gating charge.^1^

On the other hand, state-dependent accessibility of cysteines replacing some of the gating charges and using cysteine-modifying reagents showed that the S4 segment moves upwards, exposing the charged residues to the external milieu (Yang and Horn, 1995; Larsson et al., 1996). Given the origin of what is now known as the voltage-clamp fluorometry technique, Mannuzzu et al. (1996) modified the S4-cysteines mutants with fluorescent probes showing voltage-dependent fluorescent changes that parallel the voltage dependence of the gating charge displacement. These experiments provided physical evidence that the S4 is the voltage sensor.

Gating charges move through a thin hydrophobic septum at the cell membrane. Testing the voltage-dependent accessibility of the S4 residues in Na_v_ channels, Yang and Horn (1995) found that two of the S4 charges were accessible from the inside at hyperpolarizing voltages and accessible from the outside in response to depolarization. They moved across the whole electric field, transferring 1 e0 each. However, the following charge was only accessible from the inside regardless of the applied voltage. A parsimonious explanation for these results is that during channel activation, the S4 charges move through a thin hydrophobic septum of about 10 Å (Yang et al., 1996). The idea that the S4 is displaced between water-lined crevices separated by a thin septum was corroborated by replacing the S4 charges for histidine residues and probing the state-dependent accessibility to proton in a proton gradient (Starace and Bezanilla, 2001; Starace and Bezanilla, 2004). Defining the four arginines that contribute to the total Shaker K^+^ channel gating charge as R1-R4, Bezanilla’s laboratory found that when mutated to histidines, R2 and R3 behave as proton carriers implying that they move across the entire length of the electric field. Remarkably, when a histidine replaces R1, a voltage-dependent inward rectifier proton pore is produced (i.e., it conducts protons only when voltage sensors are at rest). The R4H also form a proton channel, but it conducts only when sensors are in the active position (Starace and Bezanilla, 2001; Starace and Bezanilla, 2004). Thus, all R1-R4 move from the internal to the external water-lined crevice during Shaker K^+^ channel gating, strongly suggesting that, as in Na_v_, the crevices are separated by a narrow division. Moreover, structural and electrophysiological studies indicate that the electric field is focused in a hydrophobic plug or charge transfer center of about 10 Å in thickness (Chen et al., 2010; Tao et al., 2010; Lacroix and Bezanilla, 2011). Using a fluorescent positively charged bimane derivative (qBBr) strongly quenched by tryptophan, Priest et al., (2021) show that charge displacement consists of rotation and a tilted translation during activation, but the detailed movement differs for R1 and R2.

#### 2.1.3 Electromechanical coupling

**Figure 4:**
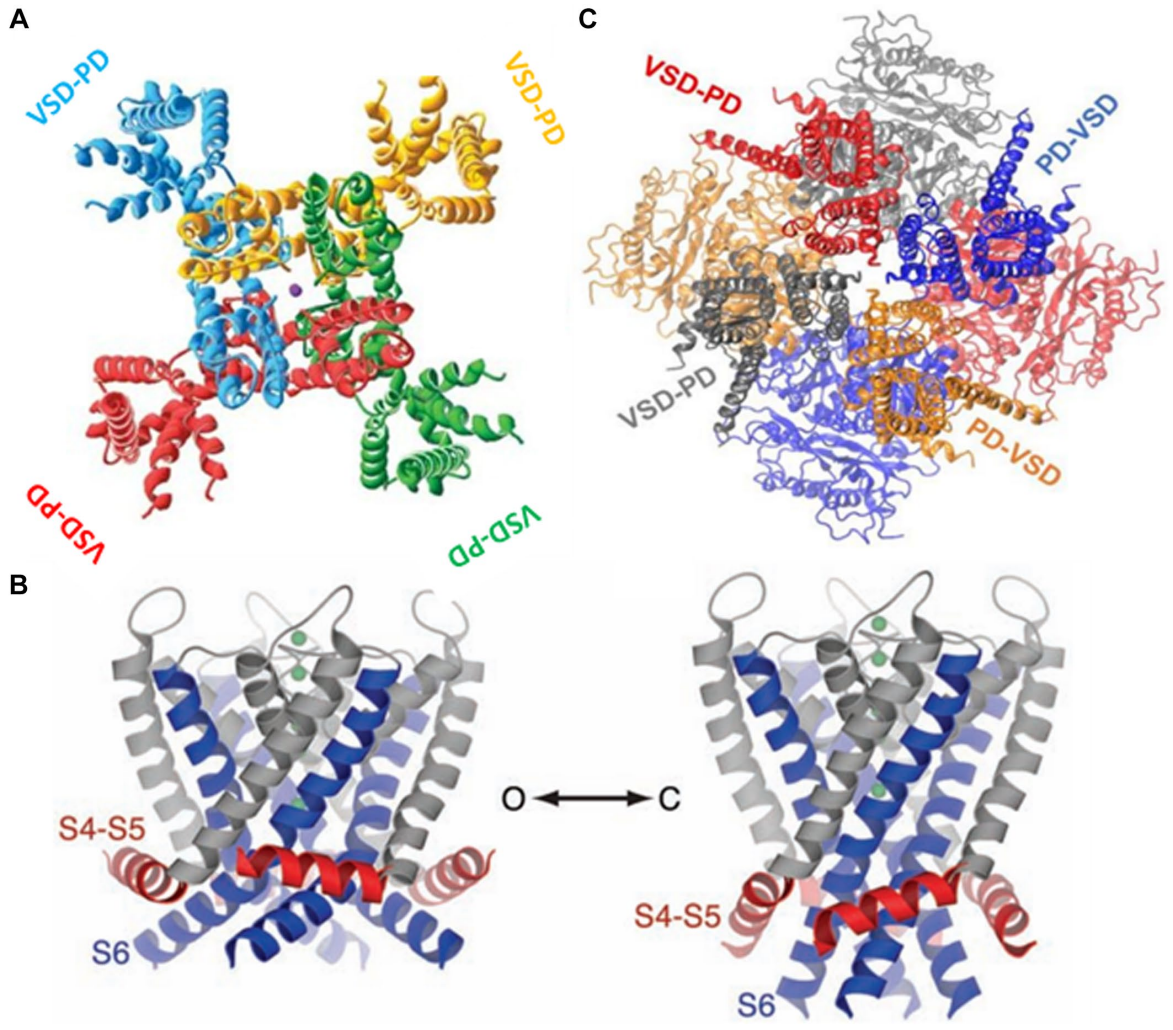
**(A)** The swapped configuration of the K_V_ 1,2 channel. Each subunit is represented in a different color. The sphere in the center is a potassium ion ([2]PDB ID 2A79). **(B)** The non-swapped configuration of the BK channel. Notice the VSD is contacting the pore of the same subunit. The c terminals in the background are shown forming the gating ring [3]. **(C)** Left. The open structure of the K1.2 channel. S6 is showed in blue and the S4-S5 linker in red. Right. A model for the closed structure of the Kv1.2 channel (Long et al., 2005b).

Now, we turn to the question of how the electrical energy contained in the displacement of the S4 is transformed into mechanical energy, leading to the pore (or ion channel) opening. The structure of K_v_ in its open-inactivated conformation (Long et al., 2005a) and Na_v_ (Shen et al., 2017) channels show they are formed by modular proteins. K_v_ channels are tetramers, and the Na_v_ protein-forming channel consists of four (I-IV) domains. Each subunit in K_v_ or domain in Na_v_ consist in a voltage sensor domain (VSD; S1-S4) and a pore domain (PD; S5-S6) (Figure 4). The structure of the K_v_1.2 shows the VSD are in a swapped configuration (Figure 4A). The VSD makes contact with the pore domain of an adjacent subunit (domain-swapped channels). Although the structure of the closed Kv channel has not been elucidated, all the results to date indicate that the four S6 transmembrane segments form a bundle crossing that hinders the passage of ions. The question is how this bundle separates to allow ion conduction during activation. The first indications regarding the coupling between the VSD and the pore opening were given by the group of Lu et al. (2001) and Lu et al. (2002), who demonstrated that the S4-S5 linker interacts with the C-terminal of S6. The structure of the K_v_1.2/K_v_2.1 (Long et al., 2007) showed that C-terminal of S6 contacts the S4-S5 linker N-terminal of the same subunit and the C-terminal of the S4-S5 linker of the S6 of the adjacent subunit. Since only the open K_v_ structure has been determined, we start with the S6 bundle crossing in its open configuration. When the S4 moves downward upon closing, the S4-S5 linker pushes the S6 toward the closed configuration of the gate (Long et al., 2005b, 2007; Jensen et al., 2012; Yarov-Yarovoy et al., 2012; Figure 4B). Above, we have described the canonical electromechanical coupling. However, several recent reports provide evidence that the interactions of amino acid residues at the interface of VSD and the pore domain contribute to electromechanical coupling in voltage-dependent channels (Fernández-Mariño et al., 2018; Kalstrup and Blunck, 2018; Carvalho-de-Souza and Bezanilla, 2019; Bassetto et al., 2021). This non-canonical coupling can play an essential role in those voltage-dependent channels lacking a well-defined S4-S5 and, particularly in those channels in which the VSD is adjacent to the P-domain of the same subunit (non-domain swapped channels). This is the case of the voltage-gated potassium channels (KCNH) family (Whicher and MacKinnon, 2016; de la Peña et al., 2018; Cowgill and Chanda, 2021) and the Ca^2+^ and voltage-activated (BK) channels (Hite et al., 2017; Tao et al., 2017; Carrasquel-Ursulaez et al., 2022; Figure 4C).

#### 2.1.4 Voltage-dependent ion channels and neuromodulation

Different types of Na_v_ channels are found in the nervous system (Na_v_1.1-Na_v_1.9) coded by nine genes (*SCNA1A-SCNA5A*; *SCNA8A-SCNA11A*) (Cox et al., 2006). Na_v_ channels can be distinguished by their sensitivity to tetrodotoxin (TTX). Nanomolar concentrations of TTX block Nav1.1-Nav1.4, Nav1.6, and Nav1.7, whereas Nav1.5 (cardiac Nav), Nav1.8, and Nav1.9 are TTX-resistant channels. Na_v_1.3, Nav1.7, Na_v_1.8, and Na_v_1.9 are involved in nociception in sensory neurons (Cox et al., 2006). Loss-of-function mutations of Na_v_1.7 have been found in families with congenital insensitivity to pain (Kaplan et al., 2016). Na_v_1.1 and Na_v_1.2 are present in GABAergic neurons of the central nervous system; missense and loss-of-function mutation of these channels create neuronal hyperexcitability and various forms of epilepsy (Kaplan et al., 2016). The most abundant Na_v_ in the brain is Na_v_1.6, and loss-of-function mutation promotes gait disorders, ataxia, and dystonia. Na_v_1.6 is enriched in the node of Ranvier of myelinated excitatory neurons and plays an essential role in initiating and propagating the action potential by lowering the threshold voltage (Wagnon et al., 2016).

After opening, Na_v_ channels inactivate [they have an inactivating gate (Liu et al., 2023)] and cannot be opened until the membrane potential is repolarized. While inactivated, neurons cannot fire another action potential, which is defined as the absolute refractory period. The absolute refractory period is followed by a relative refractory period in which sufficient Na_v_ channels are open to produce an action potential. However, the threshold current to elicit the spike is increased. Thus, depending on how fast the membrane is repolarized determines the length of the refractory period and the number of action potentials a neuron can fire per unit time. It is worth mentioning that modulatory β subunits of Na_v_ channels regulate the inactivation process. For example, β1 increases the expression, accelerates the inactivation, and leftward shifts the steady-state inactivation-voltage curve of Na_v_1.1 and Na_v_1.8 channels (Vijayaragavan et al., 2001). It has been noted that differences in expression and gating of Na_v_ channels may have significant consequences for the generation and propagation of action potentials in nociceptive neurons (Vijayaragavan et al., 2001).

Moreover, voltage-dependent K^+^ channels control the threshold, the duration, and the number of action potentials a neuron can elicit per unit time. The vertebrate family of protein-forming K^+^ channels has twelve members (K_v_1.1-K_v_12; K_v_5, K_v_6, K_v_8, and K_v_9 are gating modifiers). However, given their tetrameric structure, their splice variants (e.g., K1.1-K_v_1.8) can form heterotetramers, increasing the number of different K_v_ channels to 40 (González et al., 2012). The variety of K_v_ channels is further increased by their association with modulatory β subunits (Rettig et al., 1994).

A few illustrative examples of how the immense variety of K_v_ channels can modulate the characteristics of action potentials and, therefore, neuronal excitability are: Some K_v_ channels, like Na_v_ channels, inactivate (dubbed A-type K_v_ channels, e.g., K_v_4), but most show delayed rectifier behavior. The inactivation gate is formed by the amino terminal of A-type channels, where the first twenty amino acids bind to the internal channel vestibule once the channel opens, blocking the K^+^ flux [ball-and-chain model (Hoshi et al., 1990)]. Moreover, several K_v_ channels of the K_v_1 subfamily are delayed rectifiers but show fast inactivation currents when expressed with the K_v_β1.1 subunit (Rettig et al., 1994; Heinemann et al., 1996). These inactivating K_v_ channels control the neuron excitability in the interspike interval. The different kinetics and voltage dependence of K_v_ channels determine their differential contribution to the action potential. For example, K_v_1 activates at low depolarizing voltages, thus controlling the action potential threshold and the number of action potentials elicited by a depolarization induced by excitatory synaptic potentials. On the other hand, K_v_ channels that required larger depolarizations to open (K_v_2 and K_v_3) modulate the action potential duration and the firing pattern (Gola and Crest, 1993; Müller et al., 2007).

The interplay between K^+^ and voltage-dependent Ca^2+^ (Ca_v_) channels is crucial in modulating neurosecretion. One example that illustrates the importance of the interaction of these two classes of voltage-dependent channels is the co-localization of BK and Ca_v_ channels forming nanodomains in neurons (Gola and Crest, 1993; Müller et al., 2007). The action potentials arriving at the nerve terminal open Ca_v_ channels, and both the depolarization andCa^2+^ entry promote the opening of BK channels, which results in a decrease in the action potential duration and a fast hyperpolarization that limit the release of neurotransmitters (Shah et al., 2022).

In conclusion, the adequate functioning of the nervous system, and most particularly of neuromodulation, is possible due to the myriad of ion channels tuned by voltage. On one direction, the rise and speed of the action potential are determined by a family of Na_v_ channels, each with its own unique characteristics, while the duration of the action potential is determined and exquisitely modulated by the large family of K_v_ channels, which affects the neurotransmitter release. On the other direction, neuromodulatory inputs (detailed later on) into the distal dendrites could act to increase synaptic potential amplitudes, increase the likelihood of dendritic Na^+^ or Ca^2+^ action-potential initiation, direct action potentials to active regions of the dendrite, or simply increase action potential amplitude at the site of synaptic input, which is also crucial for neuromodulation.

## 3 Synapses and electrical properties of the membrane (*S. C. Thébault*)

To understand the mechanisms of neuromodulation, it is crucial to consider the exquisite mechanisms underlying synaptic transmission. Synaptic transmission is considered the mainstay of the functioning of the nervous system. If the latter is anatomically divided into central and PNS, the basic functioning of synapses and the fact that synaptic transmission depends on the particular electrical properties of the neuronal cell membrane are valid concepts between both systems (Purves et al., 2001b; Glasgow et al., 2019).

As a brief overview of the context in which synaptic transmission is found, it has been estimated that one neuron can receive contacts from up to thousands of other cells and similarly, any one neuron can contact up to thousands of postsynaptic cells (Herculano-Houzel, 2009). A neuron can therefore be both a presynaptic and postsynaptic cell. In addition to the axon-dendrite contact, axoaxonic and axosomatic contacts happen and shape not only the input of information to the postsynaptic cell, but also the individual contribution of the cell to the local electromagnetic field. The information input determines if neurons will fire action potential or generate subthreshold membrane potential fluctuations. These two activities are important because they form membrane potential dynamics of single neurons, which, in relationship with neuronal population dynamics and computational properties emerging from the neuronal networks *per se*, contribute to neuronal circuit activity (Johnson et al., 2019), and subsequently neuronal functions (Glasgow et al., 2019). This multi modal and spatial–temporal communication demands the same degree of complexity of neural interfaces to be used in therapies, capable of modulating such communication mechanisms.

Chemical and electrical synapses allow neurons to communicate. Neuronal contacts can be made through a gap separating the presynaptic and postsynaptic cell and/or through gap junctions, forming a channel made of connexin proteins, which mechanically link both the presynaptic and postsynaptic cell. The former contact corresponds to the *chemical synapse*, whereas the latter is known *electrical synapse*. As the name suggests, chemical synapses use chemical messenger molecules, called neurotransmitters, which are stored in presynaptic vesicles and are released to the synaptic cleft to bind receptors in the postsynaptic neuron. This is followed by the activation of downstream signaling processes and the opening of gates, known as ion channels, to allow ions to flow into the postsynaptic cell. In contrast, electrical synapses use ions and messenger proteins that can pass through gap junctions to transmit electrical impulses and molecules.

A fundamental step in chemical neurotransmission is the release of neurotransmitters to the synaptic cleft, which is dependent of intracellular Ca^2+^, known from the earliest work of Dr. Ricardo Miledi and recognized as the “Ca^2+^ hypothesis” (Miledi and Slater, 1966; Katz and Miledi, 1968; Jeng, 2002; Eusebi, 2007). Neurotransmitters can be classified in excitatory and inhibitory, *excitatory neurotransmitters* allow cation influxes, and anion effluxes to increase the likelihood of an action potential firing in the postsynaptic neuron, and *inhibitory neurotransmitters* allow anion influxes, and cation effluxes do the opposite (IFC, n.d.). It should be noted that in addition, neuromodulators released at synapses further shape synaptic transmission (O’Brien, 2014, 2019; Alcedo and Prahlad, 2020; McCormick et al., 2020; Wu et al., 2022). The development of neuromodulation therapies is closely linked to knowledge of precise changes in synaptic transmission, such as exact thresholds of stimulation, as well as the frequency and duration of pulses needed to control desired changes in targeted neural circuits. These aspects will be discussed later.

Electrical properties of the neuronal membrane are critical to understand developing neuromodulation therapies, and in the last decades these are being incorporated in the design of *in-silico* models from a single axon to the whole body, which is being used to design electrical requirements for neuromodulation (Davids et al., 2019, 2020; Stefano et al., 2021). For any synapse to function, as for any cell to live, an electrochemical gradient across the cell membrane must exist (Hammond, 2015; Levitan and Kaczmarek, 2015). It is essential for the neuron to maintain a negative transmembrane electrical potential at rest, the specific value of which depends on the type of cell. For example, in the dark, photoreceptors hold a relatively depolarized membrane potential (~ −40 mV) compared to typical neurons, which have a resting membrane potential of −70 mV (Purves et al., 2001a; Henley, 2021a). Just as crucial to neuronal responsiveness to inputs are ion channels (Hille, 1987). The neuronal plasma membrane is characterized by a large density (Beaulieu-Laroche et al., 2021) and diversity of these transmembrane proteins that allow the passive movement of ions across membranes and thus, electrical signaling in the nervous system (Hammond, 2015). The properties of ion conductance’s through neuronal cell membranes are the topics of numerous book chapters and reviews (to cite a few: Levitan and Kaczmarek, 2015; Hammond, 2015; Dolphin et al., 2020). As stated in the previous section, voltage-gated Ca^2+^ channels are crucial for neurotransmitter release, while ligand-gated channels shape the post-synaptic response.

I would like to take the opportunity to emphasize that there is more to synaptic transmission than the classical neurophysiology of Na^+^, K^+^, and Ca^2+^ channels shaping action potentials (Levitan and Kaczmarek, 2015), as well as post-synaptic potentials (Burke and Bender, 2019; Henley, 2021b). As previously mentioned, synaptic transmission relies on the single neuron transmembrane potential, which is influenced by local field potentials that initiate from regulated ion channel activity (Nolan, 2016; Sinha and Narayanan, 2022), and if chemical synaptic activity is considered to be the main contributor to local field potentials (Herreras, 2016), intrinsic ionic conductances that support the existence of intrinsic oscillation generators (Whittington et al., 2018), electrical synapses, and slow fluctuations of the membrane potential of glial cells are equally important factors in the local variations of electric fields. All the above put the synaptic transmission and its modulation at the heart of neuronal communication, which demands the same level of complexity of neural interfaces to be able to decode and modulate the neuronal function. Examples of how neuromodulation underlies the flexibility of neural circuit operation and behavior by modulating synapses, synaptic strength and dynamics, and neuronal excitability have been previously detailed [for reviews, see Deer and Constant (2014), Nadim and Bucher (2014), Bazzari and Parri (2019), Pacholko et al. (2020)]. Our understanding of the modulation of the activity of neural circuits should also include the study of local variations in electric fields at the level of tissues or organisms, with a particular focus on the role of magnetic fields generated or not generated by neuronal electrical activity.

## 4 Neurotransmitter switching: electrical activity regulates neurotransmitter identity in health and disease (*M. Pratelli, N. C. Spitzer*)

Before artificially modifying the activity of neuronal circuitry as occurs in Bioelectronic Medicine, we need to understand its function during natural conditions. Understanding brain development, as well as adult plasticity, may help us to decipher the highly complex self-modulation of neuronal circuitry. Beyond millisecond signaling, electrical activity acts on slower time scales to regulate the assembly and maintenance of neurons throughout the lifespan. It achieves this through coupling to cell metabolism (e.g., gene expression, protein synthesis). Spontaneous activity is essential during development to regulate neurogenesis, neuronal differentiation, programmed cell death, migration, myelination, establishment of proper connectivity, and synaptic pruning (Luhmann et al., 2016; Faust et al., 2021; Pumo et al., 2022). In the adult brain, electrical activity maintains and regulates the number, shape, and receptor composition of synapses, as well as neurotransmitter identity in behaviorally relevant circuits (Spitzer, 2017; Pan and Monje, 2020). Our lab has long been interested in activity-dependent regulation of neurotransmitter identity in different animal models. Electrical activity affects neurotransmitter identity during development and in adults.

During early development, spontaneous electrical activity generates Ca^2+^ transients that guide neuronal differentiation. Using the frog *Xenopus laevis* we showed that increasing the frequency of Ca^2+^ transients in neurons of the developing spinal cord increases the number of GABAergic inhibitory neurons as well as the expression of the GABA synthetic enzyme, GAD67 (Spitzer et al., 1993; Gu and Spitzer 1995). Suppressing or enhancing Ca^2+^ transient frequency in the neural tube stage *Xenopus* embryos increases the number of neurons expressing excitatory or inhibitory neurotransmitters, respectively (Borodinsky et al., 2004). Remarkably, changes in neurotransmitter identity are accompanied by matching changes in the type of receptors expressed postsynaptically, allowing the formation and maintenance of functional synapses (Borodinsky and Spitzer, 2007; Hammond-Weinberger et al., 2020). It is therefore no surprise that changes in the type of transmitter expressed influence animal behavior (Dulcis and Spitzer, 2008; Demarque and Spitzer, 2010).

**Figure 5:**
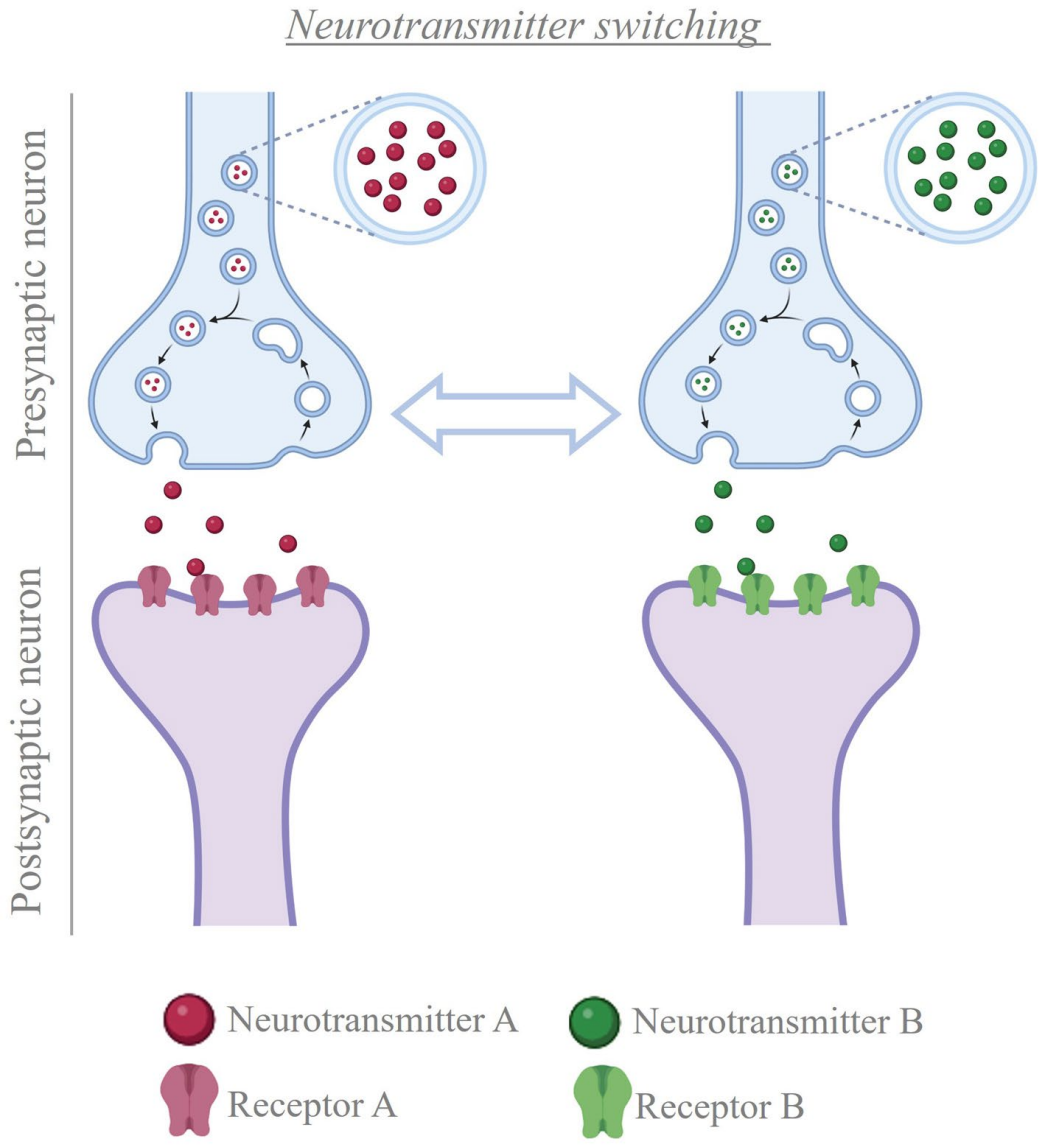
Neurotransmitter switching. Cartoon depicting the process of neurotransmitter switching. In the presence of a stimulus, neurons can change the transmitter expressed, from the transmitter they were expressing before to a new transmitter (e.g. from neurotransmitter A to neurotransmitter B, or vice versa). This change in neurotransmitter is accompanied by matching changes in the population of postsynaptic receptors, thus altering the signal that the presynaptic neuron conveys to the post-synaptic cells. Created with BioRender.com.

Interest in exploring the plasticity of neurotransmitter in the adult mammalian brain led to recent studies focused on showing how electrical activity regulates neurotransmitter plasticity. Multiple external stimuli that increase neuronal activity in specific brain regions cause changes in the type of transmitter expressed by local neurons in mice and rats (Gutiérrez, 2000; Gutiérrez, 2002; Dulcis et al., 2013; Spitzer, 2017; Meng et al., 2018; Romoli et al., 2019; Li and Spitzer, 2020; Porcu et al., 2022; Pratelli et al., 2022), with corresponding changes in postsynaptic receptors (Dulcis et al., 2013; Figure 5). However, when exposure to the inducing stimulus is coupled with experimentally-driven suppression of neuronal hyperactivity, the change in transmitter identity is prevented (Gutiérrez, 2000; Gutiérrez, 2002; Dulcis et al., 2013; Spitzer, 2017; Meng et al., 2018; Romoli et al., 2019; Li and Spitzer, 2020; Porcu et al., 2022; Pratelli et al., 2022). Once the change in transmitter has occurred, neuronal activity helps stabilizing the newly acquired phenotype. Experimentally-driven manipulation of electrical activity can successfully reverse the change in transmitter identity after it has occurred, rescuing the linked behavioral alterations (Pratelli et al., 2022). These findings open new opportunities when designing therapies where modulation of neurotransmission is required.

### 4.1 Changes in neurotransmitter identity can be beneficial or detrimental

A main question in neuromodulation is whether treatment will achieve beneficial or detrimental results. Activity induced-changes in neurotransmitter identity can substantially impact the ability of the individual to deal with the environment, by promoting either advantageous or disadvantageous changes in behavior. Beneficial effects have been linked to changes in the transmitter identity of midbrain neurons and could pave the way for novel treatment opportunities in motor and neurodegenerative disorders (Table 1; Steinkellner et al., 2018, 2022; Li and Spitzer, 2020; Lai et al., 2023). In contrast, changes in the neurotransmitter phenotype of hypothalamic neurons cause stress-induced behaviors in response to seasonal changes in photoperiod length (Dulcis et al., 2013; Porcu et al., 2022). Examples of maladaptive changes in neuronal transmitter identity are also found after prolonged exposure to drugs of abuse. In multiple brain regions, different addictive drugs cause alterations in the number of neurons expressing specific neurotransmitters (Grieder et al., 2014; Kesby et al., 2017; Ladrón de Guevara-Miranda et al., 2017; James et al., 2019; Romoli et al., 2019), some of which have been linked to drug-induced changes in behavior (James et al., 2019; Romoli et al., 2019; Pratelli et al., 2022). Activity-dependent changes in neurotransmitter phenotype linked to generalized fear and anhedonia have also been observed in the dorsal raphe in response to acute and chronic stress, respectively (Li et al., 2023).

**Table 1:** Neurotransmitter switching and its behavioral effects.

Original transmitter phenotype (A)	Novel transmitter phenotype (B)	Stimulus	Brain region	Behavioral effect	References
ACh	GABA	Voluntary running	PPN	Enhances the acquisition of motor skills	Li and Spitzer (2020)
GABA	GABA+DA	Chronic nicotine exposure	SN	Reduces motor deficits and α-synuclein expression in a mouse model of Parkinson's	Lai and Dulcis (2020) and Lai et al. (2023)
DA	DA+Glut	Exposure to toxic insults	VTA and SN	Increases neuronal resiliency to insults	Steinkellner et al. (2018) and Steinkellner et al. (2022)
DA	SST	Change in photoperiod length	PVN	Anxiety- and depression-like behaviors	Dulcis et al. (2013)
NMS	VIP	Change in photoperiod length	SCN	Not tested	Porcu et al. (2022)
Glut	DA	Neonatal nicotine exposure + adult nicotine	VTA	Increased drug preference	Romoli et al. (2019)
Glut	GABA+ (low Glut)	Repeated exposure to phencyclidine (PCP) or methamphetamine (METH)	PL	Cognitive deficits	Pratelli et al. (2022)
Glut	5-HT	Chronic stress	DR	Anhedonia	Prakash Stark et al. (2020)
Glut+5HT	GABA+5HT	Acute foot shock stress	lwDR	Generalized fear	Li et al. (2023)

Abbreviations: ACh, acetylcholine; GABA, γ-aminobutyric acid; PPN, pedunculopontine neurons; DA, dopamine; SN, substantia nigra; Glu, glutamate; VTA, ventral tegmental area; SST, somatostatin; PVN, paraventricular nucleus of the hypothalamus; NMS, Neuromedin S; VIP, vasoactive intestinal polypeptide; SCN, paraventricular nucleus of the hypothalamus; PL, prelimbic cortex; 5-HT, 5-hydroxytryptamine (i.e. serotonin); DR, dorsal raphe; lwDR, dorsal raphe lateral wings.

### 4.2 Opportunities for non-invasive manipulation of electrical activity to achieve health benefits

Appreciation of the extent to which activity-dependent changes in neurotransmitter phenotype influence behavior raises enthusiasm for the possibility of achieving therapeutic outcomes by leveraging neuronal activity via non-invasive stimulation methods. DBS and transcranial magnetic stimulation (TMS) allow modulation of electrical activity in the human brain and are already showing promising results in clinical settings. However, the underlying mechanisms of action remain unclear. Further spatiotemporal therapies are being developed. Changes in transmitter expression have been observed after both DBS and TMS (Trippe et al., 2009; Volz et al., 2013; Makowiecki et al., 2018), enhancing the potential use of these approaches for targeted manipulations of neurotransmitter expression. However, we are still far from being able to selectively modulate the activity of specific neuronal subtypes using these approaches. Development of non-invasive stimulation methods with higher cell-type specificity, as well as an increased understanding of the circuits and mechanisms driving activity-dependent changes in transmitter expression, are needed for optimized therapeutic intervention. The use of focused ultrasound for transcranial neuromodulation appears promising, as this approach can achieve high spatiotemporal precision and reach neurons located in deep regions of the brain (Rabut et al., 2020). This calls for a collaborative and multidisciplinary effort to promote the development of novel and optimized methods of non-invasive electrical manipulation, as well as the individuation of suitable neurobiological targets for therapeutic interventions.

In conclusion, fostering the development of effective Bioelectronic Medicine requires, among other efforts, a better understanding of how electrical activity affects behavior by causing changes in neurotransmitter identity. Ideally, experimental efforts should proceed on two levels. On one hand, it is important to understand under which circumstances experience-induced changes in neuronal activity affect neurotransmitter identity, causing behavioral alterations. This can provide valuable targets for alleviating specific symptoms via Bioelectronic Medicine. On the other hand, research should also aim to unveil the effects that different brain stimulation approaches have on neurotransmitter identity. Such knowledge can be useful not only to optimize therapeutic interventions, but also to identify potential off-target effects of brain stimulation that could lead to side effects.

## 5 Glial cells and neuromodulation (*A. Verkhratsky, M. E. Tremblay*)

Neuroglia play a fundamental role in neuromodulation through comprehensive control over brain homeostasis and multiple functions, including neuronal synapses formation, activity and plasticity (synaptic pruning, stripping, neurotransmitter production and recycling), neuronal metabolism (supplying neurons with metabolic substrates), blood–brain and brain-meninges barriers (astroglial and microglial endfeet forming glia limitans perivascularis and superficialis), and immune responses (phagocytosis, release of inflammatory mediators). Neuromodulation therapies increase the capillary permeability, which likely modifies astrocytic and microglial endfeet, but could also have consequences at sites of contact with synapses and other parenchymal elements. The implant of neural interfaces triggers inflammation and instigates the formation of astrocytic barrier (erroneously referred in the literature as ‘glial scar’) and fibrotic scar around the implant, which directly interfere with the performance of both recording and stimulation neural interfaces due to the increase of resistance between electrode-neurons (see Section 7). In the neuromodulation field, the concepts of astrocytes and glia or neuroglia are often used interchangeably, which deserves clarification. It is worth mentioning that not all neuroglia are astrocytes, and astrocytes are not the only glial elements interacting with an implanted electrode as part of the foreign body response. Microglia as well as cells of the oligodendroglial lineage also need to be taken into consideration.

Neuroglia are non-neuronal and (mostly) non-excitable cells of the nervous system, which are the ultimate supportive elements responsible for maintaining homeostasis of the nervous system, for providing myelination, and for mounting defense and neuroprotection. Through multiple homeostatic pathways neuroglia play an important role in regulation of neuronal excitability, synaptic plasticity, ion homeostasis, and energy metabolism. Moreover, neuroglia are key players in the neuronal immune system having a direct impact on a pathogenesis of all neurological disorders, including neuropsychiatric and neurodegenerative diseases, such as amyotrophic lateral sclerosis (ALS), epilepsy, Parkinson’s disease (PD), Alzheimer’s disease (AD), and frontotemporal dementia (FTD) (Bazzari and Parri, 2019; Pacholko et al., 2020; Šimončičová et al., 2022; Vecchiarelli and Tremblay, 2023). Thus, in neuromodulation therapies neuroglial cells are modified and also respond to the neuronal modifications. At continuation (In this section), we present main insights of neuroglia and define concepts with relevance in the field of neuromodulation that is so crucial for Bioelectronic Medicine.

### 5.1 Neuroglia: definition and classification

Neuroglia are an extended class of cells populating peripheral and central nervous systems (PNS and CNS respectively) responsible for homeostatic support and defense of the nervous tissue (Verkhratsky and Butt, 2023). Neuroglia of the PNS are represented by Schwann cells (myelinating, non-myelinating, perisynaptic and subcutaneous), satellite glia residing in sensory and autonomic nervous system ganglia, and enteric glia that provide physiological support for the enteric nervous system (Verkhratsky and Butt, 2023). Neuroglia of the CNS are classified into macroglia (astroglia, wrongly referred as astrocytes in the neuromodulation field (see Section 5.3), oligodendroglia, and microglia. Regarding the embryonic/developmental origin, neuroglia of the PNS and macroglia of the CNS are of neuroectodermal origin (PNS neuroglia originate from the neural crest, whereas macroglia from neuroepithelial cells of the neural tube); microglia are of mesodermal origin, being the scions of fetal macrophages that enter and populate the neural tube in the early embryonic development (Verkhratsky and Butt, 2023). Neuroglia emerge early in the evolution (Verkhratsky and Nedergaard, 2016); the very first neuroglial cells shape and support peripheral sensory organs (known as sensilla) and provide homeostatic support to neuronal ganglia and primitive CNS in invertebrates (Verkhratsky et al., 2022). Microglia cells show remarkable evolutionary conservation of their morphology, function and genetic signatures from leech to human (Geirsdottir et al., 2020). All neuroglial cells constantly communicate with other glia, with neurons and the vasculature; such tightly coordinated communications are central for the integration of all cellular elements into the active milieu of the nervous tissue, and underlie its functional activity (Escartin et al., 2021; Lim et al., 2021).

**Figure 6:**
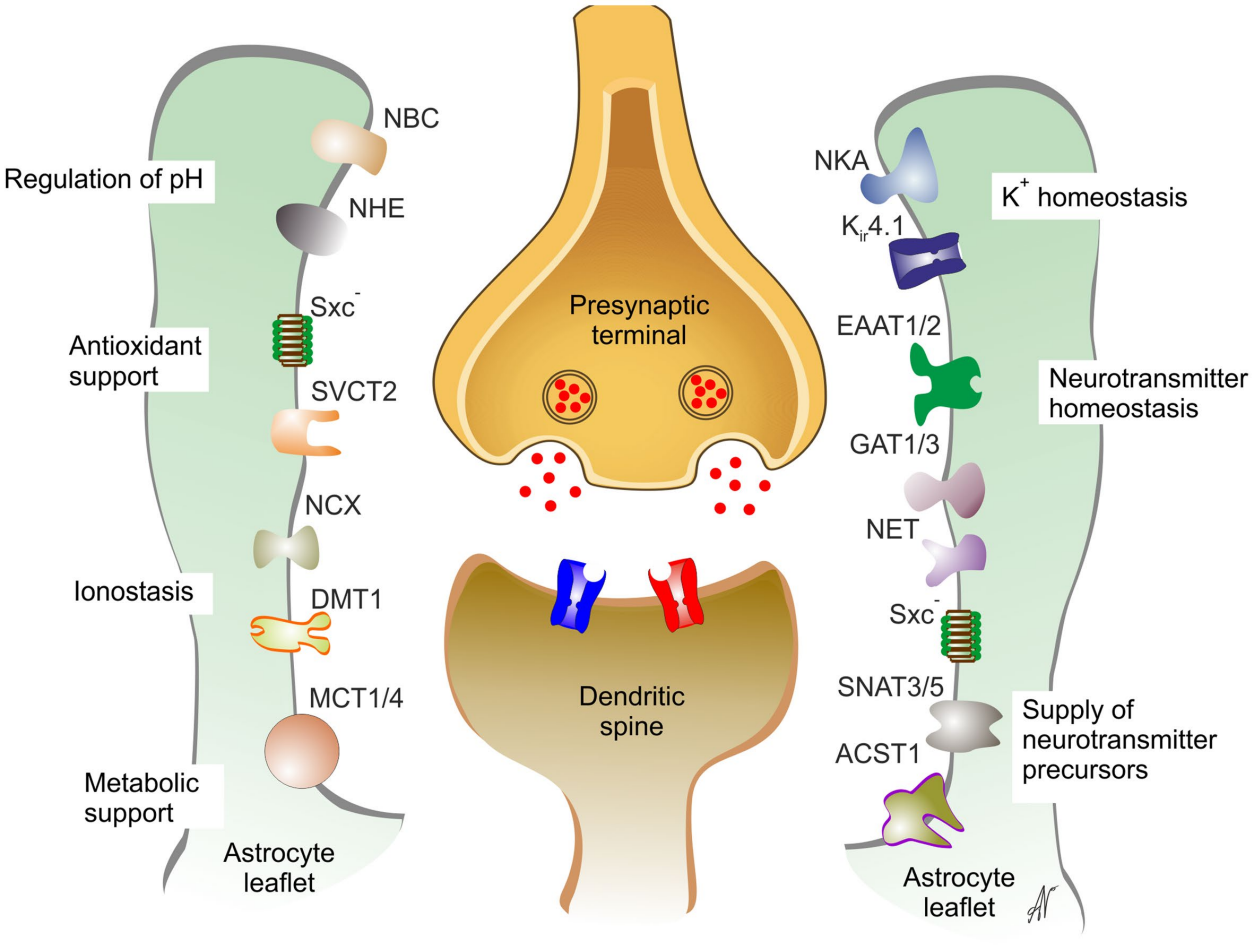
Astrocytes support and maintain synaptic transmission. DMT1, divalent metal ion transporter 1; EAAT1/2, excitatory amino acid transporter 1,2; GAT1/3, GABA transporters 1,3; Kir4.1, inward rectifier K+ channel 4.1; MCT1/4, monocarboxylate transporters 1,4; NBC, NBC1, Na+- HCO3- exchanger 1; NCX, Na+-Ca2+ exchanger; NET, noradrenaline transporter; SNATNHE, Na+-H+ exchanger; NKA, Na+-K+ ATPase; SNAT3/5, Na+-coupled neutral amino acid transporter 3,5; SVCT2, Na+-dependent vitamin C transporter 2; Sxc-, glutamate/cystine exchanger. Reproduced from Verkhratsky and Butt (2023).

The homeostatic function of neuroglia is executed at all levels of organization from organ to organism. Radial glia are the universal neural progenitors, while in developing brain nascent glial cells (microglia, astroglia and oligodendroglia) provide the migratory pathways for neural cells, shape the nervous system cyto-architecture, regulate synaptogenesis and synaptic remodeling through synaptic pruning, and provide for myelination of axons thus securing connectome. Astroglia and oligodendroglia regulate ionostasis and homeostasis and turnover of many essential neurotransmitters and neurohormones; some astroglial cells act as a central chemosensors thus being involved in systemic homeostatic control. Astrocytes, which are a subtype of astroglia (see Section 5.3), form the parenchymal part of blood–brain barrier and regulate the glymphatic system CNS-wide clearance (Figure 6). Finally neuroglial cells, and microglia in particular, are ultimate elements of the brain defense which define preservation of the nervous tissue, resolution of pathological processes and repair (Verkhratsky and Butt, 2023).

### 5.2 Ionic signaling as a substrate for glial excitability

Neuroglial cells (with exception of some oligodendroglia precursor cells) are electrically non-excitable (i.e., they are unable to generate regenerative action potentials upon electrical stimulation). Nonetheless, all neuroglial cells actively respond to incoming signals; the substrate for glial excitability is represented by spatially and temporally controlled fluctuations in concentration of intracellular ions, which regulate and control numerous molecular cascades contributing to glial physiological functions. Intracellular excitability of astrocytes is also supported by second-messengers which are controlled by multiple types of metabotropic receptors (Verkhratsky and Rose, 2020; Verkhratsky et al., 2020a,b). Multiple types of ion channels, receptors and transporters, which are operational in glial cells, generate transmembrane ionic fluxes in response to physiological stimulation. These ion fluxes translate into spatio-temporally organized ionic signals, which, in turn, regulate activity of multiple targets such as cytoplasmic enzymes or plasmalemmal transporters regulating glial homeostatic responses (Verkhratsky and Rose, 2020). Neuroglia of the PNS, as well as astroglia, oligodendroglia and microglia possess an elaborated Ca^2+^ signaling system; Ca^2+^ signals regulate gene expression, various metabolic processes, secretion and cell motility; in pathological contexts Ca^2+^ signals trigger evolutionary conserved defensive programs known as reactive gliosis (Alberdi et al., 2013; Brawek and Garaschuk, 2017; Tay et al., 2019; Escartin et al., 2021; Lim et al., 2021). Astrocytes respond to neuronal activity by generation of substantial and long-lasting Na^+^ signals, mainly associated with Na^+^ influx through glutamate transporters, the latter being the main pathways for clearing glutamate released in the course of synaptic transmission. Astrocytic Na^+^ signals coordinate astrocytic homeostatic responses (mediated mainly by Na^+^-dependent transporters) with neuronal activity (Rose and Verkhratsky, 2016; Verkhratsky and Rose, 2020). Astrocytes also possess elaborated molecular machinery for controlling movements of Cl^-^; release of astrocytic Cl^-^ sustains inhibitory transmission in the CNS (Untiet et al., 2023).

### 5.3 Functions of astroglia

Astroglia are a class of neural cells responsible for homeostasis of the CNS. Astroglia include many cell types, heterogeneous in form and function. It is important to note that astrocytes are only a part (however large) of astroglia and these terms are not interchangeable: *all astrocytes are astroglia but not every astroglial cell is an astrocyte* (Verkhratsky and Butt, 2023). Astroglia include radial glia of the developing brain, parenchymal astroglia (represented by many classes of astrocytes, such as, for example, protoplasmic astrocytes of the grey matter, fibrous astrocytes of the white matter, or velate astrocytes of cerebellum), radial astrocytes such as Bergmann glia of cerebellum, Müller cells of the retina, radial stem astrocytes of the neurogenic niches, and ependymoglia, tanycytes and choroid plexus cells lining the walls of the ventricles (García-Cáceres et al., 2019; Verkhratsky and Butt, 2023; Yeh et al., 2023). Higher primates and humans possess several unique forms of astroglial cells such as interlaminar astrocytes or astrocytes with varicose projections (Colombo and Reisin, 2004; Oberheim et al., 2009). Parenchymal astroglia and especially protoplasmic astrocytes are arguably the largest group, which regulate homeostasis of the CNS. At the same time, popular notion of astrocytes being the most numerous glia is incorrect; in humans, astrocytes account for –0–40% of all glia depending on the brain region (Verkhratsky and Butt, 2023). The term ‘astrocyte’, invented by Mikhaly von Lenhossék (Michael, 1895) literally means star-shaped cell (αστρον κψτοσ; *astron, star, kytos, a hollow vessel,* later *cell*); which reflects appearance of these cells when stained with Golgi technique. In reality, most of astrocytes are characterized with complex, spongiophorm morphology, defined by high density of terminal tiny processes known as leaflets (Lim et al., 2021; Semyanov and Verkhratsky, 2022). In that, defining astrocytic morphology as star-like (which is quite frequent in the literature) is incorrect. Among main functions of astrocytes are (i) maintenance of ionostasis, (ii) control over neurotransmitters’ clearance and turnover, (iii) metabolic support, (iv) erection of the CNS barriers in the form of glia limitans, (v) shaping of the neuro-glio-vascular unit, and (vi) formation and regulation of the operation of the glymphatic system (Verkhratsky and Nedergaard, 2018; Verkhratsky and Butt, 2023).

Concentration of ions in the interstitium is of paramount importance for maintaining neuronal excitability, and astrocytes are the main regulators of ionic movements that define ionostasis of the nervous tissue (Verkhratsky and Nedergaard, 2018). Astrocytes buffer extracellular K^+^ concentration affected by K^+^ extrusion accompanying neuronal activity. In the neuropil, most of the K^+^ is released from synaptic structures linked with dendritic arborizations and spines, whereas axonal action potentials account for a smaller fraction of K^+^ entering the interstitial space (Howarth et al., 2012; Shih et al., 2013). At the synaptic level astrocytes provide for K^+^ buffering, by accumulating K^+^ during neuronal activity and releasing K^+^ after the cessation of the latter. Clearance of K^+^ into astrocytes is mainly mediated by Na^+^/ K^+^ pump, which, in astrocytes, contains α2 subunit sensitive to extracellular K^+^ concentration (D’Ambrosio et al., 2002; Larsen et al., 2014). Release of K^+^ from astrocytes occurs through K_ir_4.1 channel (Chever et al., 2010).

Another fundamental function of astrocytes is the maintenance of homeostasis and synthesis of neurotransmitters. Astrocytes are the main producers of glutamate; neurons cannot synthesize glutamate *de novo* (Andersen et al., 2022). At the same time astrocytes remove the bulk of glutamate released during synaptic activity. Both newly synthesized and accumulated glutamate are converted into glutamine by astrocyte-specific enzyme, glutamine synthetase. Glutamine in turn is released from astrocytes and accumulated by through Na^+^-dependent transporters; this glutamine subsequently is converted into glutamate, which is either utilized in inhibitory synapses or further converted to GABA to be used in inhibitory neurotransmission. This cycle is known as glutamate/GABA-glutamine shuttle (Danbolt, 2001; Hertz and Rothman, 2016; Andersen et al., 2021). Glutamate clearance by astrocytic glutamate transporters (known as excitatory amino acid transporters 1 and 2, or EAAT1/2) is of critical importance for sustaining neuronal function; failure of astrocyte glutamate clearance instigates excitotoxicity and is one of the main pathological mechanisms of neuronal death in neurological diseases. Besides glutamate astrocytes remove GABA and glycine through dedicated Na^+^-dependent transports, and act as a central element of catabolism of monoamines being the main possessors of monoaminoxidase-B in the CNS (Verkhratsky and Butt, 2023). Astrocytes also supply neurons with L-serine, the obligatory precursor of D-serine, which acts as a co-agonist of neuronal NMDA receptors (Wolosker et al., 2016).

Astrocytic peripheral processes known as leaflets (Escartin et al., 2021; Lim et al., 2021) closely contact central synapses forming, together with microglial processes and extracellular matrix the so-called multipartite synapse (Verkhratsky and Nedergaard, 2018; Aramideh et al., 2021; Lim et al., 2021). Astrocytic compartment is indispensable part of synaptic connectivity in the CNS; astrocytes regulate synaptogenesis, synaptic maturation, synaptic isolation, synaptic maintenance and synaptic extinction thorough multiple mechanisms formalized in the concept of astroglial synaptic cradle (Verkhratsky and Nedergaard, 2014). Through multiple transporters densely populating the membrane of the astrocytic perisynaptic leaflet, astrocytes regulate all aspects of synaptic transmission and control homeostasis on the synaptic cleft (Figure 6).

### 5.4 Functions of oligodendroglia

Oligodendroglia are myelinating cells of the CNS. Oligodendrocyte lineage cells include myelinating oligodendrocytes, oligodendrocyte precursor cells (OPC) and satellite oligodendrocytes. Myelin is an extension of the oligodendrocyte cell membrane that forms concentric lamellae around axons to provide electrical insulation and facilitate rapid saltatory conduction of action potentials (Nave and Werner, 2014; Verkhratsky and Butt, 2023). Adaptive myelination of the CNS axons lasts throughout the lifespan and represents an important mechanism of neuroplasticity (Pease-Raissi and Chan, 2021). Postnatal CNS contains specific pool of adult OPCs with highly idiosyncratic properties; many of these adult OPC are electrically excitable, many receive synaptic inputs and some form synapses with adjacent neurons (Yi et al., 2023). Recently, OPCs were also shown to play various physiological roles independently from oligodendrogenesis and axonal myelination (Buchanan et al., 2023). For instance, OPCs contribute to synaptic remodeling via phagocytosis, a role previously attributed to astrocytes and microglia exclusively, in response to sensory experience during neuronal circuit refinement, both over the course of postnatal development and adulthood (Auguste et al., 2022). In addition, microglia were revealed to modulate OPCs and their functions. In particular, microglia determine OPC number by phagocytosing them during early postnatal development, which has functional outcomes on the process of axonal myelination (Nemes-Baran et al., 2020).

### 5.5 Functions of microglia

**Figure 7:**
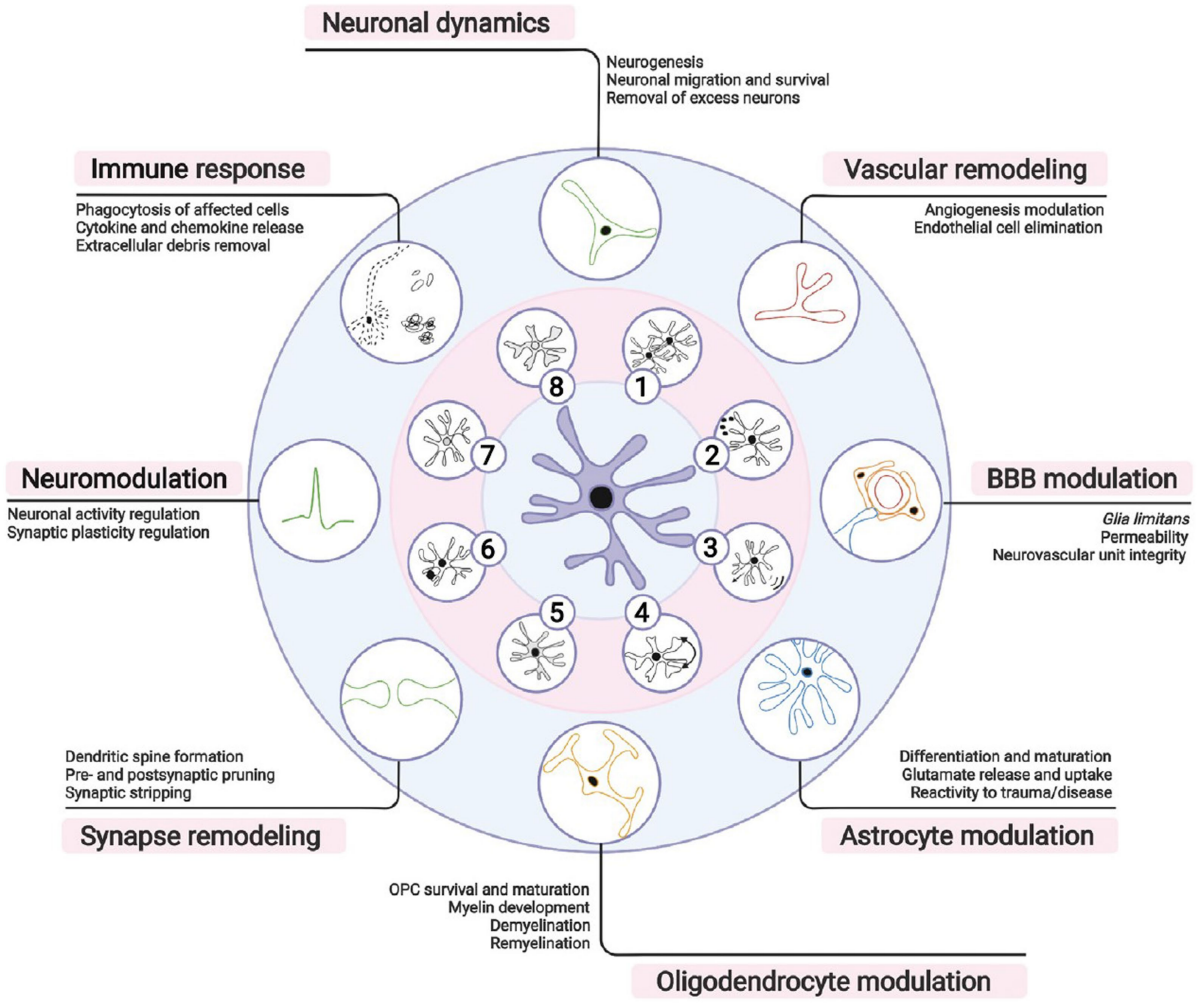
Microglial importance for brain functioning. Microglia, the brain resident immune cells, fulfill a broad repertoire of activities beyond immunity in both health and disease. According to local microenvironmental cues, microglia can (1) proliferate, (2) secrete soluble factors, (3) migrate, or (4) adapt their morphology, (5) metabolism, (6) phagocytic activity, (7) transcriptome, and (8) proteome. During brain development, microglia participate in neuronal migration and survival by stimulating neurogenesis or removing excess neurons. Microglia similarly contribute to modulating blood vessel formation and endothelial cell elimination during vascular remodeling. Postnatally, microglia form part of the glia limitans of the blood– brain barrier (BBB), actively regulating its permeability and repair. In the brain parenchyma, microglial function requires an intimate crosstalk with astrocytes necessary also for astrocytic development, immune responses and/or reactive states. Moreover, microglia are needed for proper oligodendrocyte progenitor cell (OPC) survival and maturation, myelination, and myelin turnover throughout life. Synaptic remodeling, crucial for learning and memory, is achieved via microglial partial (i.e., trogocytosis), or full (i.e., phagocytosis) pruning, displacement of synaptic elements (i.e., stripping), and formation of dendritic spines via release of neurotrophic factors. Neuronal activity and excitability are influenced by regulation of short- and long-term synaptic plasticity via release of neurotrophic factors, cytokines, or neuromodulators. During immune responses, microglia coordinate peripheral immune cell infiltration and clear debris, apoptotic or infected cells. It is important to note that many of these responsibilities are shared with neurons, astrocytes, oligodendrocytes or border-associated macrophages, whose concerted actions are required for homeostasis. Reproduced from Šimončičová et al. (2022)_._

Microglia are the innate immune cells of the CNS. They are traditionally known for their roles in clearing toxic debris, removing dysfunctional or degenerating cells, as well as pathogens, and for mediating inflammation. However, over the past decade, microglia were also found to contribute to neuronal dynamics, vascular remodeling, as well as blood–brain barrier, astrocyte, and oligodendrocyte modulation. Microglia additionally partake in synapse remodeling, axonal myelination, neuromodulation, and immune responses (Figure 7). These various beneficial physiological functions of microglia are overall important for CNS development, maturation, activity, plasticity and integrity, but also behavior and cognition across the lifespan (Paolicelli et al., 2022; Šimončičová et al., 2022).

Recent multi-omics studies have identified key signatures expressed by microglia that support the view of their heterogeneity, a concept which was first described using light and electron microscopy approaches (Stratoulias et al., 2019), and currently holds the potential to unravel their functions across contexts of health, injury and disease (Tremblay, 2021). Microglial transcriptomic signatures indicate a diverse cellular population comprised of many states that vary according to the spatiotemporal context (Vecchiarelli and Tremblay, 2023). This diversity is most pronounced during development, when microglial physiological functions are extensively recruited, as well as upon disease or injury (Vecchiarelli and Tremblay, 2023). A next step of major importance for the field will be to identify the functional roles performed by the multiple emerging microglial states, in interaction with the other neuroglia and neurons, with the aim to target them therapeutically (Stratoulias et al., 2019; Tremblay, 2021; Paolicelli et al., 2022; Vecchiarelli and Tremblay, 2023).

A microglial role that has significantly changed the microglia field is their involvement with synaptic pruning, which refers to the elimination of synaptic elements. Different cellular and molecular mechanisms can be recruited, including phagocytosis which can be complete or partial (and then named trogocytosis, a form of nibbling), as well as synaptic stripping (referring to the physical separation of pre- and post-synaptic elements by intervening microglial processes) (Vecchiarelli and Tremblay, 2023). This synaptic pruning process is activity- and experience-dependent, contributing to the refinement of neuronal networks during critical periods of postnatal development as well as into adolescence and adulthood. Microglial remodeling of neuronal circuits also mediates the adaptation of the brain and behavior to the living environment. Several molecular mechanisms involved in synaptic pruning were identified, including the classical complement pathway, fractalkine signaling, and TREM2 (Šimončičová et al., 2022).These mechanisms involved in synaptic pruning can take a pathological turn upon injury and disease, notably leading to synaptic loss which is a strong pathological correlate of cognitive decline during aging and in neurodegenerative diseases, supporting the importance of their therapeutical targeting (Šimončičová et al., 2022).

Beyond synaptic pruning, microglia can release various trophic factors, such as the brain-derived neurotrophic factor (BDNF), which can contribute to CNS development, maturation, activity, plasticity, and integrity in many ways. For instance, microglial trophic factors can increase neuronal arborizations, numbers of synapses, promote neurogenesis, as well as synaptic plasticity throughout various stages of life, including development and adulthood. Microglial BDNF-mediated formation of synapses was notably shown to be required for synaptic plasticity and learning (Parkhurst et al., 2013). Neurogenesis is also a key process in the developing and mature brain which is important for learning and memory, as well as stress responses and emotional regulation (Cameron and Glover, 2015), and can become impaired in several psychiatric disorders and neurodegenerative diseases, as well as following injury, across the lifespan. Microglial role in neuroplasticity mediated by trophic factors like BDNF can additionally result in desirable anti-depressant and anxiolytic effects, and cognitive recovery upon disease and injury (VanderZwaag et al., 2023). Emerging evidence is further indicating that promising pharmacological treatments for mood disorders, such as psychedelics, could produce their therapeutic efficacy by specifically acting on these microglial mechanisms (VanderZwaag et al., 2023).

### 5.6 Neurogliopathology

The neuron-centric doctrine that shaped neuropathological thinking for the main part of the 20^th^ century, has been challenged in recent decades with an increased notion of the fundamental role of neuroglia in defining the progression and outcome of all neurological diseases (Verkhratsky and Butt, 2023). Pathological changes in neuroglia are complex and include (i) defensive reactive gliosis (astrogliosis, microgliosis and reactivity of oligodendroglia), (ii) neuroglial atrophy and loss of function, (iii) neuroglia degeneration and death (represented for example by clasmatodendrosis for astrocytes and microthanatosis for microglia), and (iv) various forms of gliopathies with the emergence of aberrant glial forms that drive the neuropathology (Pekny et al., 2016; Verkhratsky et al., 2017; Sofroniew, 2020; Escartin et al., 2021). Different pathological glial phenotypes may co-exist in the same pathological process or can be associated with different stages of the disease or disorder. Reactive gliosis is an evolutionary conserved complex program of biochemical, physiological and structural changes triggered by the lesions to the nervous tissue. Reactive gliosis is prominent in traumatic, infections, ischemic and auto-immune lesions to the CNS (Sofroniew, 2014). Reactive astrocytes, microgliocytes and OPCs migrate to the lesion and ultimately form the glial barrier (glia limitans perilaesiones) between fibrotic scar and normal tissue; reactive glia also support post-lesional regeneration (Faulkner et al., 2004; O’Shea et al., 2022). Neuroglia atrophy and loss of function is a characteristic feature of many neurological diseases including neurodegenerative, neuropsychiatric and neurodevelopmental diseases; failed homeostatic support represents one of the main mechanisms of neurotoxicity. In summary neuroglia are the main target for new therapeutic strategies that may be a game-changer in finding the cure for diseases of the CNS.

In conclusion, neuroglia areessential for the homeostasis of nervous tissue. They maintain the coherence of the nervous tissue, including the brain, retina, and spinal cord, as an organ, and contribute to determining the systemic outputs of the nervous system and thus to cognitive functions and behaviors. Neuroglia is considered to be the fundamental player in neuropathology that determines the progression and outcome of all neurological disorders.

## 6 Systems neuroscience (*S. C. Thébault, M. A. González-González*)

Systems neuroscience is a specialized field within neurosciences, it is dedicated to unraveling the function of neuronal assemblies and how these are integrated as a system to produce measurable behavior (included but not limited to cognitive, somatic or autonomic, and sensorial or motor) (Gerstein et al., 1989; Rizzolatti et al., 2018). In Bioelectronic Medicine, the design of high-precision therapies requires a medullary understanding of how neurons behave when connected to form neuronal pathways and large networks in the brain and in the periphery (Pavlov and Tracey, 2022). The ideal therapy in Bioelectronic Medicine is the one delivered to a specific target without recruiting undesired ones, thereby mitigating off-target effects. This level of precision is achieved by both, the anatomical area of implantation and the validation of electrical parameters that elicit the desired physiological effect (Pavlov et al., 2020; Pavlov and Tracey, 2022). Thus it becomes fundamental the dialog between technology developers and neurophysiologists to define well informed bioelectronic outcomes.

**Figure 8:**
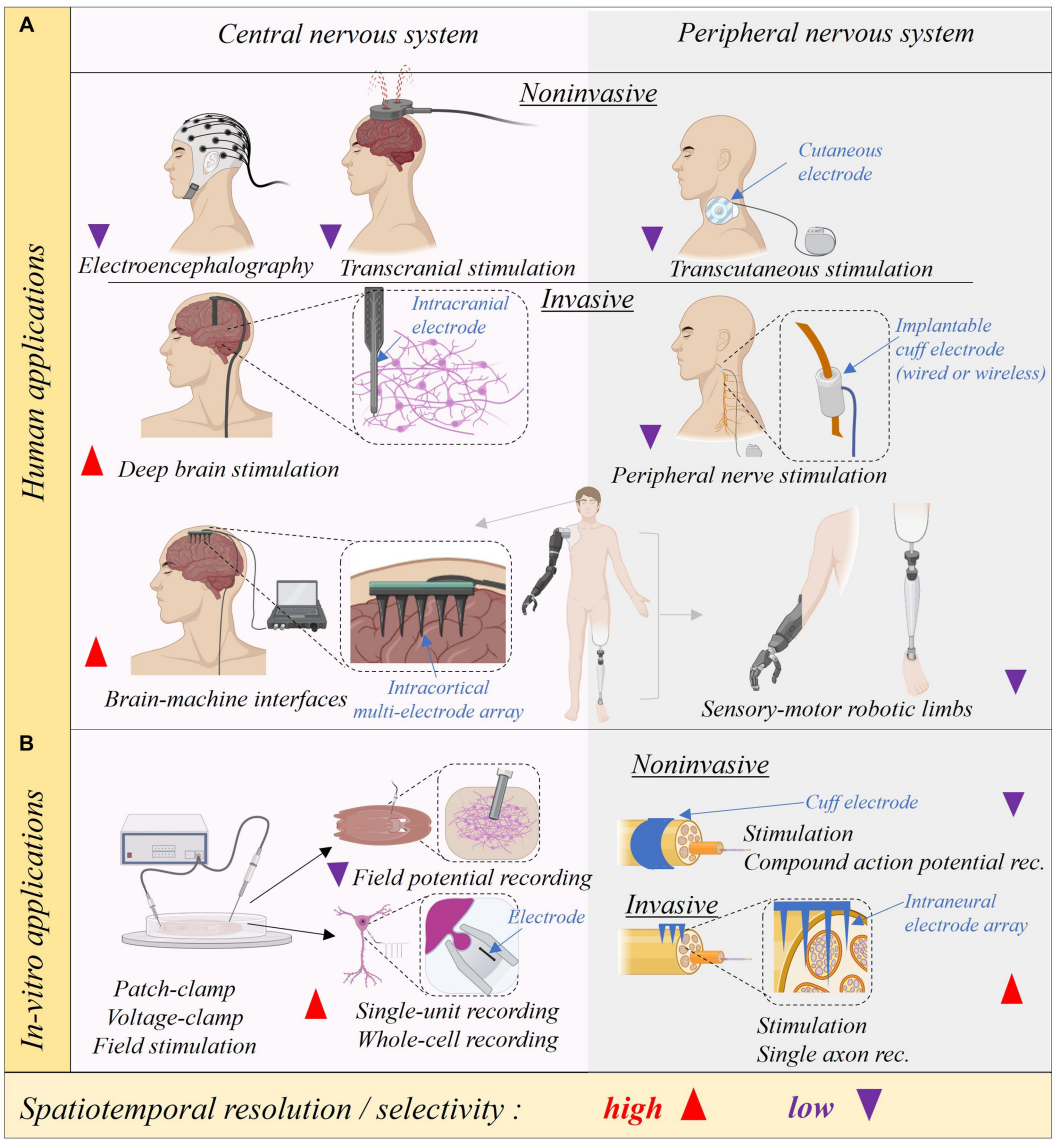
Spatiotemporal resolution of neural interfaces. The use of neural interfaces can be separated in those used in central and peripheral nervous system and according to its invasiveness*. In-vivo* applications for use in humans are summarized in **(A)** and *in-vitro* applications are summarized in **(B)**. Spatiotemporal resolution for recordings or for stimulation selectivity is coded with red or purple arrows for high and low resolution, respectively. Created with BioRender.com.

Organisms encompass different nature of neuronal circuits encountered across many spatial and temporal scales (Luo, 2021). To decode and modulate both CNS and PNS, from single channel to the neuronal population dynamics, neural interfaces require the right spatiotemporal resolution for both, recording and stimulation. The highest spatiotemporal resolution has been achieved *in-vitro* (i.e., whole cell and single channel patch clamp recordings) and *in-vivo* with invasive neural interfaces, which has motivated the development of new technologies in miniaturized, less invasive, and more biocompatible and reliable neural interfaces. Examples of several approaches of different nature and spatiotemporal resolution are displayed in Figure 8.

In the last decades, a considerable amount of resources have been devoted to understanding the brain circuitry, yet less attention has been invested in unveiling the peripheral neural code as a system. Recognizing the inherent complexity of studying these systems, initiatives, such as the International Brain Initiative (IBI) (The Lancet Neurology, 2021) and the NIH BRAIN initiative (Hsu et al., 2020) have emerged. These promote the development and use of quantitative resources to understand brain circuits from cellular to behavioral scales and across multiple species. Some outcomes include progress in the neuromodulation of the spinal cord for motor recovery after stroke (Powell et al., 2023), DBS personalization in the treatment of resistant depression, and the identification of neuronal signatures predicting pain in the brain of patients with chronic pain, by the use of intracranial electrodes (Shirvalkar et al., 2023). This is a continuously growing field, which demands the integration of multiple disciplines, and has led to the creation of consortiums for data management to understand the nervous system. An example is the Collaborative Research in Computational Neuroscience (CRCNS) (Teeters et al., 2008), a joint program of the National Science Foundation (NSF) and NIH created to identify and implement common data science tools and resources to integrate theoretical and experimental neuroscience through collaborative research projects between theorists and experimentalist scientists.

Finally, in the field of systems neuroscience, the the progress in emerging technologies of neural interfaces plays a pivotal role in achieving high spatiotemporal resolution on the study and modulation of neuronal circuits, without compromising the performance over time in chronic implants. This is a challenging endeavor that is discussed in further sections.

## 7 Neural interfaces (*A. G. Hernández, M. A. González-González*)

Neural interfaces are devices that interact with the nervous system that aim at decoding neural function or modifying its activity (Hatsopoulos and Donoghue, 2009; Valeriani et al., 2022). Different classifications have been used taking into account the invasiveness, the design, the technology, and the application (Cho et al., 2020; Redolfi Riva and Micera, 2021; Valeriani et al., 2022) (see Figure 8). In the fabrication of neural interfaces, the use of substrates and conductive materials is fundamental. A desired material is one that is biocompatible and biostable over time, while maintaining stable electrochemical properties and evoking minimal foreign body response (Hatsopoulos and Donoghue, 2009; Redolfi Riva and Micera, 2021). The implantation often evokes foreign body response, increasing inflammation and inducing the formation of an astrocytic barrier between the conductive material and the nervous tissue (CNS or PNS) (Christensen et al., 2014; Carnicer-Lombarte et al., 2021). This barrier increases the impedance and demands an increase in current for stimulating electrodes to achieve electrical thresholds that modify the neuronal activity, and for recording electrodes reduces the sensitivity (Leventhal and Durand, 2004; Carnicer-Lombarte et al., 2021). These events, lead to the ionization of the material due to the surpass of the oxide/redox capabilities of the conductive surface, which reduces its capability, may induce inflammation due to the release of chemical species, and conduce to interfacing failure (Cogan, 2008; Cogan et al., 2016). Materials technology is in continuous development, and in this section, we raise awareness of the need not only to develop neural interfaces, but also to validate them functionally according to the very complex necessities of the nervous system.

### 7.1 Electrochemical properties of conductive materials

Neural interfaces require the use of conductive materials with unique electrochemical properties (Cogan, 2008; Wu et al., 2021), which require consideration on whether the goal for the interface is neural stimulation or recording (Cogan, 2008; Harris, 2021; Vatsyayan and Dayeh, 2022). The relevance of validating electrochemical properties of neural interfaces relies on the risk of tissue damage due to harmful reactions while applying current for stimulation (Vatsyayan and Dayeh, 2022). In the last decades, the conductive materials that have gained attention due to their chemical stability and high performance are the nanostructured carbon composite materials, including nanofibers, carbon nanotubes and graphene oxide, among others (Geim, 2009; Tian et al., 2014; Thompson et al., 2015; McCallum et al., 2017; Wang et al., 2019; Gonzalez-Gonzalez et al., 2021). This is due to the specific electrochemical behavior and nanostructure that allows higher currents due to the large geometric surface area (Wang et al., 2019). Other materials include metals Pt, PtIr, Gold and conductive polymers like poly(3,4-ethylenedioxythiophene) (PEDOT) (Meijs et al., 2015; Dalrymple et al., 2020; Wu et al., 2021). Their electrochemical properties are measured *in-vitro* and include potentiostatic electrochemical impedance spectroscopy (EIS), cyclic voltammetry (CV), voltage transient (VT), and open circuit potential (OCP) (Cogan, 2008; Harris, 2021; Weltin and Kieninger, 2021). We will detail their main aspects because they are to be taken into account for applications in Bioelectronic Medicine and they help to understand some limitations encountered by this field of research.

#### 7.1.1 Potentiostatic electrochemical impedance spectroscopy

**Figure 9:**
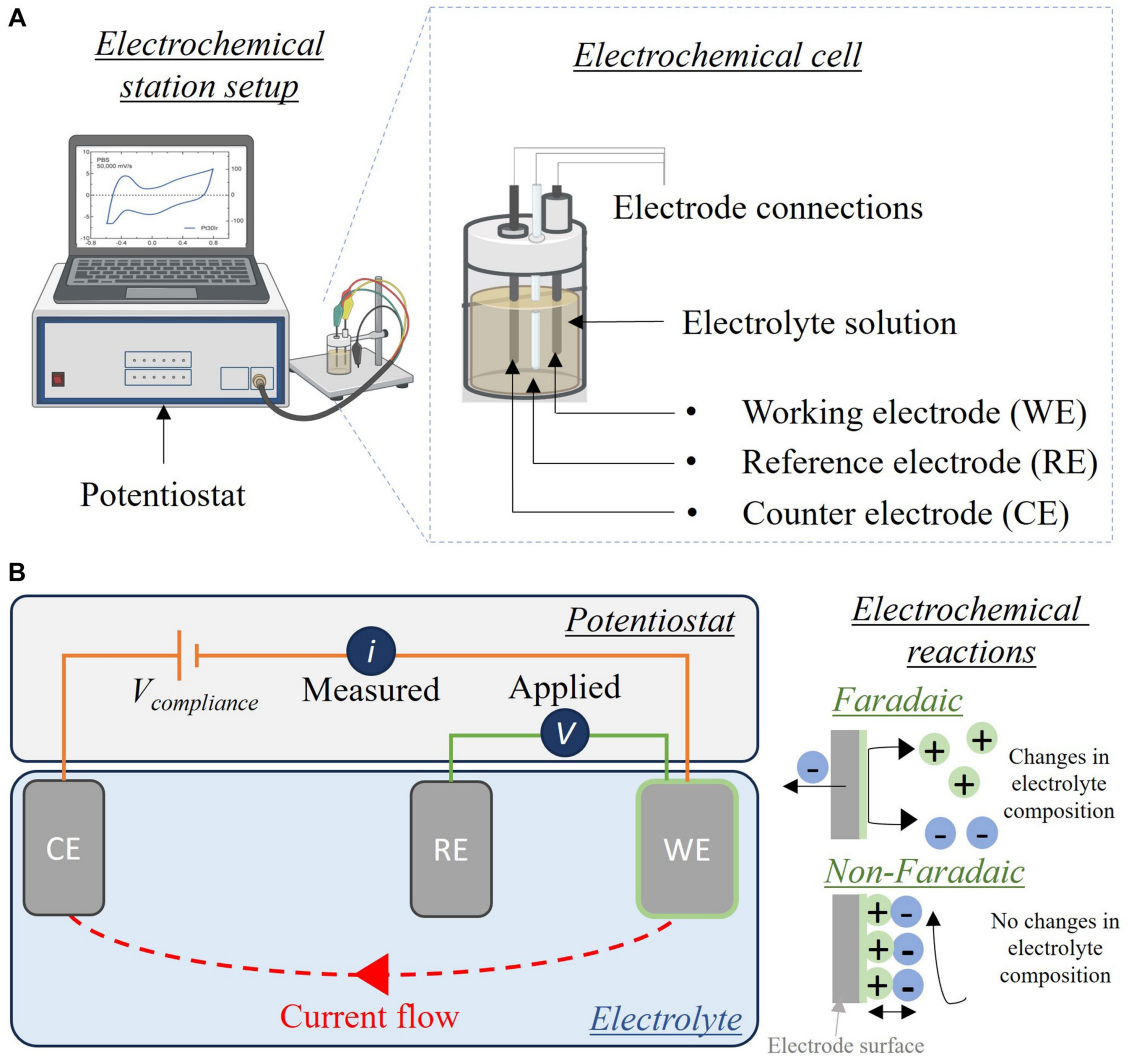
Electrochemical set up and circuit. **(A)** Electrochemical station and cell showing a three-electrode configuration. **(B)** Electrical circuit representing the electrochemical cell. In the right, Faradaic and non-Faradaic reactions representations. Created with BioRender.com.

Potentiostatic electrochemical impedance spectroscopy (EIS) is the most widely used electrochemical measurement to characterize neural interfaces (Cogan, 2008). It measures electrochemical reactions occurring at the electrode-electrolyte (or electrode-tissue) interface, including charge transfer, mass transfer, and adsorption between an electrolyte (e.g., solution or neural tissues) and the electrode surface (Magar et al., 2021; Wang et al., 2021; Weltin and Kieninger, 2021). Because of this, it is commonly used to investigate both, tissue and electrode properties. During the execution of this technique, a sinusoidal potential with frequencies ranging typically between 0.1 to 10^5^ Hz is applied between the test electrode (working electrode) and a reference electrode, having a counter or auxiliary electrode to provide input potential to the working electrode in order to complete a circuit and allow charge to flow (Figure 9). The most common material used as a reference electrode is Ag|AgCl, due to its stability in solution, its reproducible potential, and its reversibility (non-polarizability). In a reversible electrode like Ag|AgCl, a small cathodic current produces reduction, while a small anodic current produces oxidation. The counter electrode should be an inert material, tipically Pt (Jerkiewicz, 2022), with low polarization resistance to facilitate the ionic interchange (Jerkiewicz, 2022).

**Figure 10:**
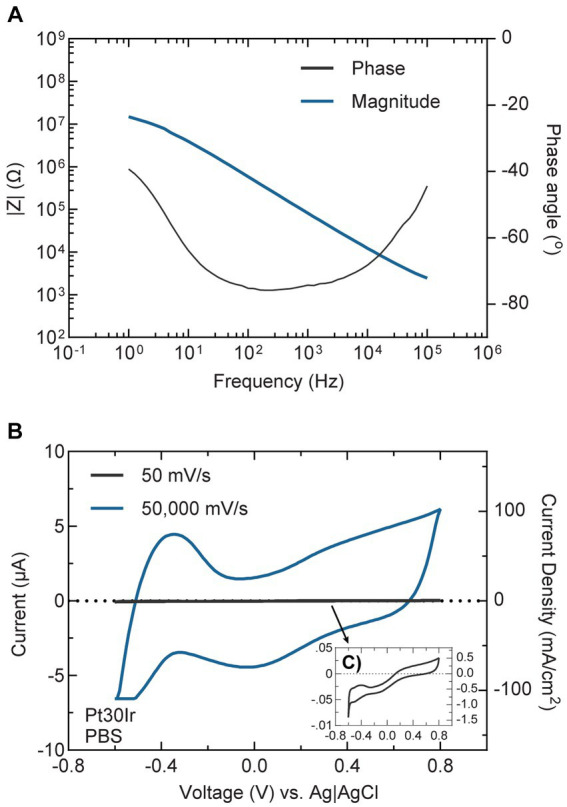
Electrochemical properties of neural interfaces. Representative electrochemical characterization of a PtIr alloy electrode (Pt30Ir), with a composition: 70% Pt and 30% Ir. **(A)** EIS of Pt30Ir showing the impedance magnitude (left y-axis, blue line) and impedance phase (right y-axis, black line). **(B)** CV voltammogram for Pt30Ir at 50 mV/s and 50,000 mV/s sweep rates. Inset **(C)** shows CV scan at 50 mV/s with left *y*-axis in μA, right *y*-axis in mA/cm^2^ and *x*-axis in V. All measurements were recorded *in-vitro*, in PBS (pH = 7.4) at room temperature following a three-electrode configuration using an Ag|AgCl reference electrode.

In EIS, the current generated between the working electrode and a counter electrode that closes the circuit is measured. The impedance magnitude and phase angle (phase shift) are calculated from the potential applied and the current measured (Figure 10). The ideal electrode may not change its properties after the test, thereby providing confidence in chronic implant stability. It should be noted that often the EIS is only performed at 10 (Cho et al., 2020) Hz, which is an edge point that informs of the chemical stability of the conductive material before reaching the electrolysis of water and an non reversible reaction harmful to the tissue (Won et al., 2018; Vatsyayan and Dayeh, 2022).

#### 7.1.2 Cyclic voltammetry

Cyclic Voltammetry (CV) is an electrochemical technique that characterizes reduction and oxidation-reaction occurring at the neural interface and informs on the reversibility of the reactions, and kinetics of electron transfer at the interface (Elgrishi et al., 2018). It helps to define parameters to maintain chemical stability in the electrode surface and to prevent non reversible oxidation or reduction during stimulation due to the generation of chemical species and electrons transfer. Such non reversible reactions are referred to as Faradaic reactions. In contrast, those reactions where charges are reversibly transferred across electrode-electrolyte interface are called non-Faradaic reactions (McCreery et al., 1988, 1990; Figure 9B). The parameters for a non-Faradaic reaction are desired in electrode implantation to promote biostability of the electrode, and are represented as the water window as shown in Figure 10. Similar to EIS, CV requires a working electrode, a counter electrode, a reference electrode, and an electrolyte (Elgrishi et al., 2018; Jerkiewicz, 2022; Puglia and Bowen, 2022). During the execution of this technique, a triangular potential waveform is applied, while measuring the current between the working and counter electrodes. The slope of the triangular waveform is known as the sweep rate and provides information regarding the potential at which electrochemical reactions occur. To avoid damage to the neural interfaces during the execution of CV, the technique should be done within the potential window of the water often named “*electrolysis water window*.” This is defined as the potential upper and lower limits at which reversible redox reactions occur (Kondo, 2018). Above the upper and lower limits of the water electrolysis window, non-reversible redox reactions occur, which could be damaging to the electrodes and contribute to the failure of the neural interface (Figure 9). In addition, useful information can be gathered from conducting CV at different sweep rates. A slow sweep rate CV provides information about the electrochemical reactions occurring along the shank of the electrode sites, while a fast sweep rate CV provides information about electrochemical reactions occurring at the surface of the exposed electrode site (Cogan, 2008). The charge storage capacity (CSC) is then measured as the area under the curve of the current measured with respect to time. It is commonly divided into two areas, cathodal (CSCc) and anodal; CSCc is the commonly reported value for the characterization of stimulating electrodes. A caveat to this, is that the electrolysis water window has been largely characterized for different electrode materials in phosphate-buffered saline (PBS), however the validity of these limits for *in-vivo* applications remains largely unknown (Vatsyayan and Dayeh, 2022).

#### 7.1.3 Voltage transients

The measurement of voltage transients is used to define the behavior of stimulating neural interfaces with a two- or three-electrode setup. It helps to define the maximum charge that can be safely delivered during a neural stimulation pulse, based on the electrolysis water window pre-determined for the stimulating material. This is defined as maximum charge injection capacity (Max Q_inj_). In voltage transients, current is injected between the working and the counter electrode, while measuring their difference of voltage (Cisnal et al., 2018). For a three-electrode setup, the voltage is measured between the working and the reference electrodes. The injected current and waveforms are defined to mimic those used for electrical stimulation of neural tissue, usually a biphasic waveform with an interphase delay. From a voltage transient, it is possible to measure the maximally cathodal-driven electrochemical potential excursion (E_mc_) during the interphase delay (Cogan et al., 2009). The E_mc_ is defined as the measured potential immediately after the cathodal pulse has ended and is used to calculate Max Q_inj_ (Cogan et al., 2007b; Cisnal et al., 2018).

#### 7.1.4 Open circuit potential

The OCP is unique for every material and in the Bioelectronic Medicine field, it helps to predict the biostability of physiological conditions. It is used to measure the open-circuit voltage (E_OC_), it defines the electrical potential of a working electrode relative to the reference electrode while no potential or electric current is applied in the electrochemical cell (Leslie and Mauzeroll, 2024). The E_OC_ is determined by the electrochemical equilibrium between the electrode and the electrolyte solution and is influenced by factors such as the composition of the electrolyte solution, or tissue biomolecules if implanted in a physiological environment. The literature reporting E_OC_ is limited in the field of Bioelectronic Medicine, but it may help to develop more reliable implantable materials.

### 7.2 Electrochemical properties of materials used in Bioelectronic Medicine

Several FDA-approved Bioelectronic Medicine applications are currently available (Kumsa et al., 2018), and additional clinical and preclinical trials are being investigated. Most of the electrodes used for delivery of electrical currents and some used for neural tissue recording in closed-loop applications, have been fabricated using the metals shown in Table 2.

**Table 2:** *In-vitro* electrochemical properties of materials used in bioelectronic medicines.

Material	Application	|Z|@1kHz (Ω cm^2^)	Electrolysis window (V)	CSCc (mC/cm^2^)	Max Qinj (mC/cm^2^)	Uses in bioelectronic medicines	References
Platinum (Pt) and Platinum/Iridium (Pt/Ir)	Stimulation/Recording	0.4–4.7	−0.6–0.8	4.4–8.0	0.1–0.3	Cochlear stimulation for deafness+, deep brain stimulation for Parkinson’s disease and essential tremor+, sacral nerve stimulation for urinary and fecal incontinence+, spinal cord stimulation for chronic pain+, etc.	Rose and Robblee, 1990; de Boer et al., (1978); Negi S et al., (2010); Fan B. et al., (2021)
Titanium Nitride (TiN)	Stimulation/Recording	0.3–4.8	−0.9–0.9	2.5–3.0	0.5–0.9	Brain-machine interfaces*, sciatic nerve stimulation and recording*, etc.	Gonzalez-Gonzalez et al., (2021); Weiland et al., (2002)
Sputtered Iridium oxide (SIROF)	Stimulation	0.1–3.8	−0.6–0.8	18–34	0.4–3.8	Intracortical microstimulation for somatosensory perceptions*, brain-machine interfaces*.	Cogan et al., (2004); Negi S et al., (2010)
Activated iridium oxide (AIROF)	Stimulation	0.1–3.8	−0.6–0.8	3.0–30	0.3–3.8	Cochlear stimulation for deafness+, intracortical stimulation for somatosensory perceptions+, occipital cortex stimulation for blindness+.	Beebe X et al., (1988) Cogan et al. (2004, 2006) Frederick et al., (2020)
Gold (Au)	Recording	6.9–33	−0.6–0.9	0.1	1.0–3.2	Motor cortex brain-machine interfaces+, sciatic nerve recordings*, hippocampal recordings*, etc.	Fan et al., (2020) Gonzalez-Gonzalez et al., (2021) Kim et al., (2013) Suyatin et al., (2013)
PEDOT	Recording	0.1–0.2	−0.9–0.6	0.8–7.0	2.3–15	Motor cortex brain-machine interfaces*, peripheral nerve recordings*.	Cogan et al., (2007a) Venkatraman et al., (2011) Hagler et al., (2022) Cui et al., (2007)
Graphene and Graphene oxide	Recording	22.5–215	−0.6–0.6	8.0–31	56–106	Peripheral motor interfaces*, In-vitro recording, bidirectional motor cortex brain-machine interfaces*.	Chen et al., (2011) Du et al., (2015)

All measurements are vs. Ag|AgCl. Data reported as range min – max; CSC reported at slow sweep rates ≤ 100 mV/s. + indicates clinical and preclinical uses, * indicates preclinical uses only.

While these values can vary based on the experimental setup, this should serve as an initial guideline for the electrochemical characterization of neural interfaces in Bioelectronic Medicine. Overall, *in-vitro* and *in-vivo* electrochemical characterizations of neural interfaces are useful in providing information about their electrical properties and stability before and after implantation, which gives hints about their performance and long-term stability.

It can be concluded from this section that the use of conductive materials with high performance and reliability for recording and stimulation of neuronal tissue is critical to the development of neural interfaces. The validation of electrochemical capabilities and biostability of these materials has enabled the investigation of safe stimulation parameters and miniaturization of the electrode sites with enhanced spatial resolution (Li et al., 2023). These advancements, alongside new technologies for data collection to optimize sample rate readouts (i.e., increasing data points per second) have not only improved the spatiotemporal resolution but also exponentially increased the size of the data collected. This improvement comes with an incredible challenge in terms of the capabilities of data handling. Furthermore, when studying simultaneously multiple biological datasets to identify their interactions, define critical therapeutic targets, and incorporate precision devices for closed-loop systems, it becomes fundamental the implementation of computational approaches in networks and artificial intelligence (AI), which are discussed in the next section.

## 8 Big data, networks, and AI in Bioelectronic Medicine (*C. G. Akcora*)

In the age of information and technology, the convergence of computational resources, big data, networks, and AI has ushered in a new era of healthcare innovation. Big data refers to extremely large and complex datasets that exceed the capabilities of traditional data processing methods and tools (Yoo and Shoaran, 2021; Basu et al., 2022; Pavlov and Tracey, 2022). The fusion of these technologies can be tailored to individual patients, fostering a new era of personalized medicine (Debnath et al., 2020; Scangos et al., 2021; Wang et al., 2022). We here report key trajectories and research areas that materialize at the crossroads of big data, networks, and AI within the domain of Bioelectronic Medicine. We delve into the potential challenges, and breakthroughs, and shed light on research questions that await exploration, guiding researchers, practitioners, and policymakers toward utilizing the full potential of this transformative field.

### 8.1 Trends and research opportunities in big data, networks, and AI in Bioelectronic Medicine

Recent advancements in big data, networking, and AI technologies are enabling unprecedented progress in Bioelectronic Medicine (Cracchiolo et al., 2021; Robinson et al., 2021). By harnessing these technologies, researchers can gather and analyze vast amounts of physiological, clinical, and environmental data to enhance our understanding of the human body’s intricate functions and diseases, and in turn develop targeted therapeutic interventions. In this matter, the National Institute of Health recently created new opportunities to propel the study of interoception, which is the mechanism to sense and regulate signals within the body (NOSI, 2023). Interoception research describes how the brain interacts with the periphery to maintain homeostasis, and if altered leads to disease, including autonomic disorders, autoimmune disease, and neurological conditions (Berntson and Khalsa, 2021). The study of simultaneous interaction of different organs, and the monitoring of multiple biomarkers in health and disease demand the use of advanced computational tools to define patterns of activity and use networks tools to identify nodes of interaction that can be translated as therapeutical targets. Furthermore, the customization of electrical neuromodulation for Bioelectronic Medicine requires reading out of physiological data to design closed-loop treatments (Ganzer and Sharma, 2019).

Intricately intertwined with this progress is the role of big data (Naeem et al., 2022)—an intricate tapestry woven from the wealth of information gathered from diverse sources, including patient health records, genomic data, and real-time physiological measurements—. Big data not only enriches our understanding of human physiology and disease, but also empowers AI algorithms to discover intricate patterns, forecast medical trends, and offer timely interventions. The synergy between big data and Bioelectronic Medicine thus not only enhances diagnostics and treatment but also propels the evolution of proactive and preventive healthcare strategies.

Furthermore, the transformative potential of Bioelectronic Medicine finds its ultimate expression through the expansive web of interconnected networks. The exchange of information between medical devices, wearable sensors, and digital health platforms creates an ecosystem that ensures real-time monitoring, data sharing, and responsive interventions. This interconnectedness not only empowers healthcare practitioners with a view of patient well-being but also empowers patients to engage in their health management journey.

At the heart of this interdisciplinary area lies AI, a technological cornerstone that breathes life into the vast data streams and interconnections. AI’s remarkable ability to decipher intricate correlations (Birkhoff et al., 2021), learn from historical data, and predict future health trajectories bolsters the precision and efficacy of bioelectronic interventions. From optimizing treatment regimens to ushering in an era of predictive medicine, AI fuels the engine of progress in Bioelectronic Medicine.

### 8.2 Big data integration and analysis

The convergence of big data collected from an array of sources, including smart devices (e.g., phones), wearable sensors, implantable devices, electronic health records (EHRs), and omics data, has propelled healthcare and medical research into a new era of possibility (Zhang et al., 2023). This approach enables the creation of an interconnected portrait of an individual’s health, encompassing genetic, physiological, and clinical dimensions. Through the application of AI-driven analytics, the data sources yield a deeper understanding of intricate relationships, unveiling patterns and connections that were previously obscured or would have required extensive clinical expertise, making it a time-consuming endeavor.

By merging genetic data with clinical records, smart device data and real-time sensor readings, healthcare practitioners and researchers gain an unprecedented perspective on how an individual’s genes, environment, and lifestyle interact to shape their well-being. This integration of diverse data streams lays the groundwork for discoveries that can transform our understanding of disease mechanisms, therapeutic targets, and predictive biomarkers.

Real-time monitoring, facilitated by wearable sensors and implantable devices, brings continuous health data into the fold (Goh et al., 2023). AI algorithms can swiftly analyze this streaming information, allowing for the early or just in-time detection of anomalies or deviations from established patterns. This proactive monitoring holds tremendous promise for swift interventions and timely medical responses, potentially altering disease trajectories and improving patient outcomes.

The synergy of big data integration and AI-driven analytics also facilitates predictive modeling. Healthcare professionals can utilize these models to anticipate disease progression, evaluate treatment responses, and tailor interventions to an individual’s unique characteristics. This shift toward personalized medicine marks a departure from the one-size-fits-all approach, enabling treatments and strategies that are finely tuned to a patient’s genetic makeup, lifestyle choices, and historical health data.

This transformative approach to healthcare is not without its challenges and bioethical considerations. The handling of vast amounts of sensitive health data demands rigorous data privacy and security measures, ensuring that individuals’ information remains protected. Furthermore, efforts must be made to mitigate biases that may be present in the data, both due to the population sampled and the technologies used for data collection. Transparent and responsible data usage practices are essential to building trust and maximizing the benefits of big data integration.

In essence, the integration and analysis of big data in healthcare offers a paradigm shift in how we perceive, monitor, and manage health. The interconnectedness of various data sources, coupled with AI-driven analytics, has the potential to uncover hidden insights, reshape medical paradigms, and drive the evolution of personalized, data-driven healthcare. As these technologies continue to evolve, the prospect of earlier disease detection, more effective treatments, and improved patient outcomes comes closer to realization.

### 8.3 Networked devices and interconnectivity

In the last 30 years, networks of interconnected physical objects or devices that are embedded with sensors, software, and other technologies to collect and exchange data over the internet or other communication networks has been studied under the umbrella term Internet of Things (IoT) (Rejeb et al., 2023). The convergence of IoT technologies with the field of bioelectronics presents a revolutionary paradigm in healthcare and medical interventions and opens up a realm of possibilities by enabling seamless connectivity between bioelectronic devices and external systems.

One of the primary advantages of integrating IoT with bioelectronics is the establishment of real-time and remote monitoring capabilities. Bioelectronic devices can now transmit vital health data to medical professionals and healthcare systems instantaneously. This real-time data exchange facilitates proactive and personalized healthcare, as medical practitioners can remotely track patients’ physiological parameters, medication adherence, and overall health status.

Furthermore, IoT-enabled bioelectronic devices empower medical professionals to make informed decisions and adjustments to treatment protocols. The ability to receive continuous streams of data allows for timely interventions and optimizations, ensuring that patients receive the most effective and personalized therapies. n critical situations, the integration of IoT and bioelectronics can be truly life-saving. Rapid response mechanisms can be established, where abnormal readings from bioelectronic devices trigger immediate alerts to healthcare providers. This real-time alert system enables swift reactions to emergencies even when the patient is not aware of a developing health risk such as a heart attack.

Collaborative networks of interconnected bioelectronic devices also hold immense promise. By pooling data from a multitude of devices, a comprehensive and detailed understanding of a patient’s health can be established. These networks can facilitate data-driven insights, leading to the development of more effective treatment strategies and therapeutic interventions. Moreover, collaborative networks can contribute to the advancement of medical research by providing researchers with large-scale, real-world data to fuel discoveries.

However, the integration of IoT into bioelectronics also introduces challenges, such as data privacy and security concerns in data and model integration across research units. Safeguarding sensitive patient information and ensuring the secure transmission of data are critical considerations in this evolving landscape. When patient data cannot be shared due to country specific laws or privacy reasons, distributed learning based models can use the data and share the model parameters with other researchers. Federated learning is such a distributed learning model that enables the training of centralized models across a network of decentralized edge devices or data sources, while keeping the raw data localized and private (Kim and Huh, 2021).

### 8.4 AI-enhanced device design and optimization

AI-powered algorithms have emerged as a transformative force in the field of bioelectronics, reshaping the way we design, optimize, and utilize bioelectronic devices (Kim and Huh, 2021). These advanced algorithms are proving to be indispensable tools in driving innovation and revolutionizing the landscape of medical therapies.

At the heart of this synergy lies the marriage between artificial intelligence and bioelectronic devices, yielding a new generation of medical technologies that are both adaptive and self-learning (Li et al., 2021). Machine learning techniques, a subset of AI, empower bioelectronic devices to transcend the traditional static models by continuously analyzing and responding to real-time patient data. This dynamic approach enables these devices to make informed, personalized adjustments to their parameters based on the individual’s unique physiological responses, thus paving the way for personalized medicine on a previously unimaginable scale.

This symbiotic relationship between AI and bioelectronics has far-reaching implications for patient care. By harnessing the power of AI, bioelectronic therapies become more than just treatments; they evolve into intelligent systems that evolve alongside the patient’s changing needs. This adaptability not only enhances the efficacy of the therapy but also contributes to a significant reduction in unwanted side effects. As AI algorithms continuously fine-tune device settings in response to the patient’s physiological feedback, the potential for achieving optimal therapeutic outcomes while minimizing adverse reactions becomes a tangible reality.

Moreover, this collaborative approach expedites the development and optimization of bioelectronic therapies. Traditionally, the process of refining and perfecting medical devices could be a laborious and time-consuming endeavor. However, AI-driven algorithms streamline this journey by rapidly analyzing vast datasets, identifying patterns, and generating insights that guide the design process. This accelerated innovation cycle translates into quicker iterations and faster translation of cutting-edge research into practical, patient-centric applications.

As AI and bioelectronics converge, the boundaries of what is achievable in medical treatment expand. Seamlessly integrating AI-powered bioelectronic devices into the fabric of healthcare systems will create efficient, precise, and patient-centered therapies. This powerful combination positions us at the forefront of a new era in medical technology, where the marriage of artificial intelligence and bioelectronics holds the potential to transform lives, redefine treatment paradigms, and inspire further breakthroughs in the relentless pursuit of improved human well-being.

### 8.5 Research opportunities

The synergy between AI and bioelectronic devices has led to a paradigm shift in medical research and treatment strategies (Mitsala et al., 2021). One pivotal application lies in predictive modeling for disease progression. Researchers can construct predictive models that closely monitor the evolution of diseases and conditions. These models offer invaluable insights by identifying key junctures for intervention and tailoring treatment approaches to thwart or decelerate the progression of these ailments. This proactive approach has the potential to revolutionize patient care, shifting the focus from reactive to preventive measures.

Delving deeper into the realm of neural pathways, advanced network analysis techniques enabled by AI are illuminating the complex web of connections within these pathways and biochemical networks. Such revelations provide a foundational understanding that is essential for designing finely tuned bioelectronic interventions. By pinpointing specific pathways, AI-driven strategies can be developed to precisely target therapeutic effects, ensuring optimal outcomes with minimal collateral impact.

Personalized medicine takes on a new dimension with the integration of AI and bioelectronics. The amalgamation of patient-specific data, encompassing genetic profiles, physiological measurements, and lifestyle information, paves the way for tailoring treatment strategies on an individual level. This is where AI truly shines, as its algorithms can process vast amounts of data to optimize device parameters and dosages unique to each patient. This personalization not only enhances the efficacy of bioelectronic therapies but also fosters a deeper connection between the treatment and the patient, acknowledging the diversity and nuances of human biology.

However, as the realms of bioelectronics and AI become increasingly intertwined, ethical and security considerations loom large. The interconnected nature of bioelectronic medicine underscores the need to address concerns surrounding patient consent, data usage, and privacy. Robust privacy-preserving techniques must be developed to ensure the protection of sensitive patient data. Additionally, the evolving landscape necessitates the establishment of comprehensive ethical frameworks that guide the responsible development, deployment, and use of AI-powered bioelectronic devices. By actively addressing these concerns, the integration of AI and bioelectronics can progress with integrity, trust, and a steadfast commitment to patient well-being.

In conclusion, the convergence of big data, networks, and AI in bioelectronic medicine promises to advance our understanding of human physiology and the development of innovative therapies dealing with large and complex biomedical data. The incorporation of a roadmap of informatics tools and computational strategies is enabling the evolution of a new era of Bioelectronic Medicine, with the aim of ultimately improving patient outcomes and thus transforming the healthcare landscape.

## 9 Biomedical materials and medical devices (*M. Ecker*)

Biomedical materials are synthetic structures, biocompatible and suitable to use as an implantable devices. In Bioelectronic Medicine these materials include substrates to pattern conductive materials, or the device body encapsulating the electronic components. In this section we will emphasize on the use of biomedical materials as a substrate in neural interfaces. In Section 7, the importance of the electrochemical validation of conductive materials was discussed, and here we will highlight the necessity of innovating on new biocompatible and biostable materials that contain the conductive materials.

**Figure 11:**
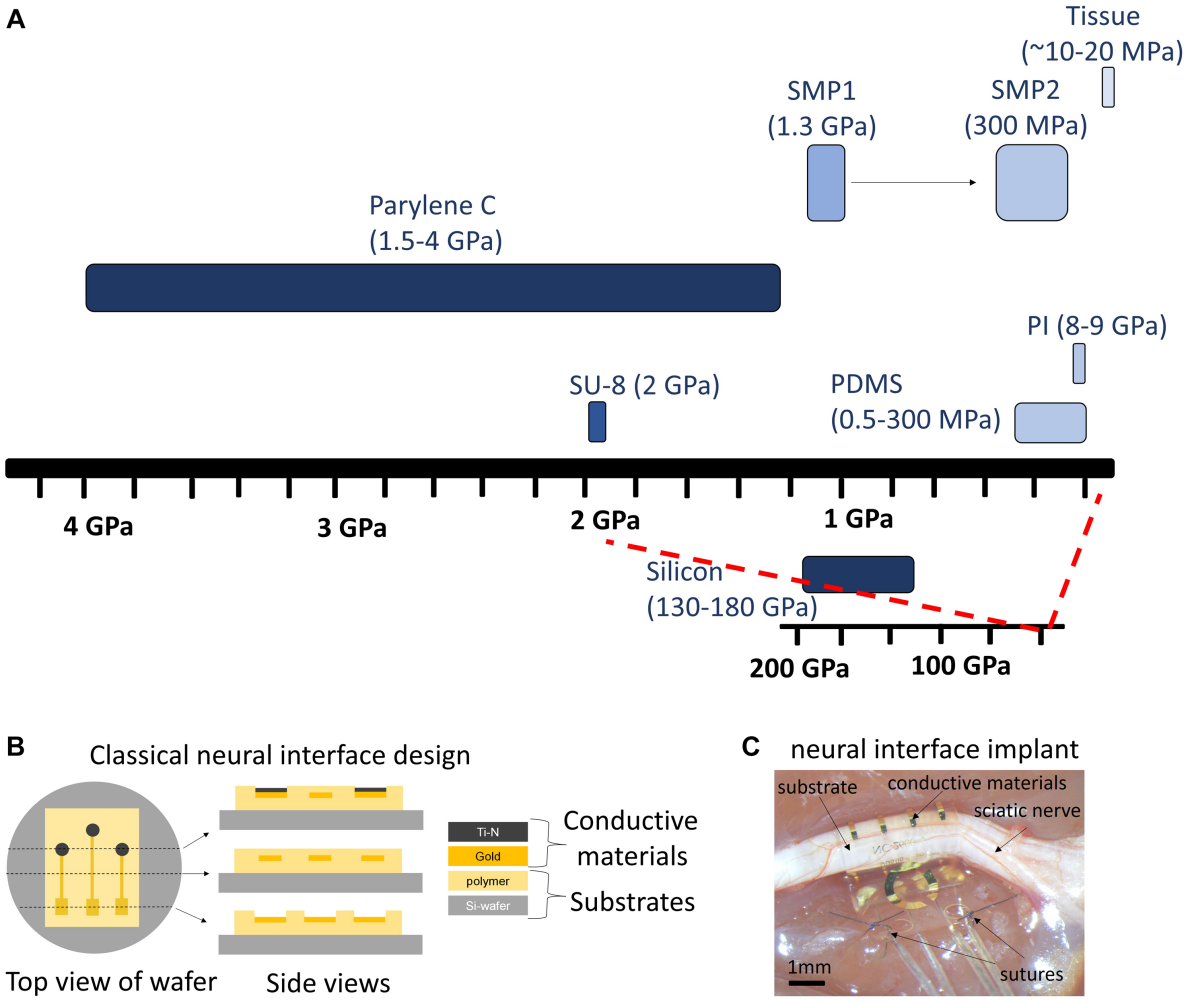
Stiffness of substrates and design-implant of typical device for PNS. **(A)** The ideal material has stiffness similar to tissue. SMP, shape memory polymer. SMP1 and 2 represent the stiffness of SMP at room temperature and at physiological temperature respectively. **(B)** Top and side views of a typical bioelectronic device for PNS. A Silicon wafer is used as a carrier substrate during the microfabrication of the device. A polymer is deposited on top of the wafer, for example by spin coating. conductive traces (Gold) and electrodes (Ti-N) are patterned and defined by photolithography. In a final step, all the conductive traces are encapsulated by an additional layer of polymer. Reactive ion etching is typically utilized to expouse the electrode sites and bonding pads. **(C)** Picture of a classical flexible shape memory polymer device implanted in the sciatic nerve of rat. Note the substrate and conductive materials adapting the surface of the nerve.

A main effort is being devoted to the development of biomimetic materials, by improving their mechanical, physical and chemical properties (Someya et al., 2016; Bettinger et al., 2020; Redolfi Riva and Micera, 2021; Kim et al., 2023). An integral part of neural interfaces consists of conductive materials (in contact with the neuronal tissue) with recording or stimulation functions. These conducting materials are typically patterned on substrates by the use of specialized processes (i.e., photolithography or bio 3D printing) (Kipke et al., 2008). The substrate is a critical component as it provides the foundation for the electronic components of the device, acts as an encapsulant, and determines the biocompatibility, biostability and flexibility of the device (Kipke et al., 2008). A typical stack of materials is displayed in Figure 11 (McCreery et al., 1988; Cogan et al., 2007b; Cogan, 2008; Cogan et al., 2016).

The ideal substrate material for bioelectronics is biocompatible, biostable, and with mechanical properties that mimic the neural tissue. These properties minimize the initiation of the immune response (foreign body response), in the CNS represented as astrocytic barrier (Moshayedi et al., 2014; Sohal et al., 2016), and in PNS as fibrotic tissue around the nerve implanted (Christensen et al., 2014; González et al., 2018; Carnicer-Lombarte et al., 2021). The mechanical mismatch (neural interface-tissue) is a main contributor to inflammatory responses, which relies on the mechanical properties of the material implanted with higher stiffness than the soft neural tissue (i.e., Young’s modulus) (Barrese et al., 2013; Moshayedi et al., 2014; Figure 11). The development of materials with low Young’s modulus, closer to the values for neural tissue, has been demonstrated to minimize the inflammatory response (Nguyen et al., 2014; Potter et al., 2014; González et al., 2018). This has motivated the development of new materials that better meet the neuronal tissue mechanics. Figure 11A displays Young’s modulus for neural tissue in contrast with different materials used in the fabrication of neural interfaces.

Traditionally, bioelectronic devices have been made of silicon as a substrate, due to its low cost and thereby, large cost-effective production. Silicon is a semiconductor, biocompatible and biostable material that is well-suited for electronic applications (Drake et al., 1988; Campbell et al., 1991). It has been used for decades to fabricate transistors, diodes, and integrated circuits, so it is well known in the industry for manufacturing silicon-based bioelectronic devices. However, silicon is a stiff and brittle material, far from the mechanical properties of the soft nervous tissue (Figure 11A), which makes it not well suited for these purposes, where flexible and soft materials are preferred to prevent inflammation and foreign body response (Barrese et al., 2013; Kozai et al., 2015; Cody et al., 2018).

In recent years, the use of polymers, ceramics, and composites has offered a new alternative to improve the mechanical properties of substrates (Won et al., 2018; Araki et al., 2020; Song et al., 2020). These materials include SU-8 (Cho et al., 2008; Altuna et al., 2012; Kim et al., 2017), polydimethylsiloxane (PDMS) (Kim et al., 2017; Baslan et al., 2021; Lienemann et al., 2021), polyimide (PI) (Vomero et al., 2020, 2022; Rodrigues et al., 2020) and poly(chloro-p-xylylene) (Parylene C) (Kim et al., 2020; Steins et al., 2022). The advantages of these materials in contrast with silicon, are the softness and flexibility (low Young’s modulus), biostability and biocompatibility, which enable applications as surface devices like electrocorticogram arrays (ECoG) or nerve cuffs for peripheral nerves (Balakrishnan et al., 2022; Witham et al., 2022). Furthermore, the feasibility of fabrication and customization for specialized needs (i.e., intracortical devices, visual prosthesis, etc.) make polymers an excellent choice for a new generation of neural interfaces.

A drawback of polymeric bioelectronics (referred to as organic) is that their manufacturing process is often more complex than for inorganic devices, which can add to the cost and make it more challenging to scale up production (Someya et al., 2016). Additionally, polymers are more susceptible to harsh chemical conditions and high temperatures., Traditional fabrication methods are not suitable, and additional process development is required to create reliable devices (Chitrakar et al., 2022). Another complication with polymer-based devices is their water absorption property, which causes swelling and changes in electrical and mechanical properties. This can lead to delamination, leakage currents, and corrosion of the metal traces (Onken et al., 2021; Oldroyd and Malliaras, 2022), which disrupts the electrical connections and compromises their use for chronic applications (Tintelott et al., 2022).

Ultimately, the choice of the material depends on the application. Penetrating devices, such as cortical probes, deep brain stimulators, interfascicular, or intraneural devices, offer high spatiotemporal resolution and precision (see Section 6, Figure 8), but require stiffer materials that can penetrate the tissue, which leads to foreign body response and compromises the chronic implantation (see Section 7). A new generation of bioelectronic devices includes the use of shape memory polymeric substrates (SMP) (Capadona et al., 2012; González et al., 2018; Stiller et al., 2020). These materials are stiff enough to allow the implantation of the device, but decrease in stiffness after implantation in response to physiological temperature and moisture (González et al., 2018; Stiller et al., 2020; Figure 11C). Other approaches are to use insertion shuttles to aid the insertion of soft and flexible probes, however it becomes traumatic for the tissue (Lind et al., 2010; Capadona et al., 2012; Shoffstall et al., 2018) In the PNS, the use of cuff electrodes demands the use of materials that are flexible, bendable, and compliant to be implanted and that meet the mechanical properties of different nerves (Witham et al., 2022; González-González et al., 2023). These properties can be modified in polymers by changing the chemical composition, the processing conditions, or the post-processing treatment.

This is an ongoing active research field that makes it possible to tailor the properties of polymers to the specific requirements of a neural interface. As technology continues to develop, the expectation is to have more innovative and function adaptable polymers that are not only long-lasting and high-efficient, but also that comply with strict translational regulatory guidelines. The path for regulatory approval is discussed in the next section.

## 10 Path to regulatory approval of a medical device (*J. Coates*)

To achieve regulatory approval for use in humans of a medical device, it is needed to first determine the path for the regulatory submission. Guidelines vary by country and there are many markets for bioelectronic medicines with diverse paths. Here, we will particularly refer to the path for approval in the USA. This includes at minimum the identification of the Class of the device (FDA, 2017), the approval pathway, and the product code (IDE, n.d.). By identifying these items, the applicable regulations can be defined, including testing requirements: in-bench, animal studies, clinical trials, and the path to first in-patient testing (IDE, n.d.), and ultimately FDA approval.

Once the applicable regulations are determined, it is a manner of aligning the design and testing process to these regulations. In some cases, particularly in novel medical devices, one applicable regulation or list of regulations does not cover all important functional and safety aspects needed for appropriately testing the device. If it is the case, it is required to generate and prove their measures of effectiveness, and then evaluate their ability to meet those and safety aspects, which include electrical and mechanical safety, biocompatibility, sterilization, biological and chemical safety, among others.

With the regulatory path information identified by specifying the country of desired marketing, and in the USA the class of device, approval pathway, product code to include applicable references and guidance documents and information gathered from technology, market, and user investigations the development process can start. An example is: “The device will be marketed in the USA and approved through the FDA as a Class III medical device following the DeNovo pathway as a product code OHA for *a heart valve, more than minimally manipulated allograph*.” Most development processes need to follow a gated, stepwise process. The specifics can vary between regulatory bodies, companies, etc., but in general the 5 steps are:

1. Develop requirements for the device (market, regulatory, user requirements)
2. Specify what those requirements mean (engineering requirements and specifications)
3. Design the device (design details)
4. Evaluate the device against the specific requirements (verification testing)
5. Evaluate the device against the measure of a successful device (validation testing)

The development team is typically made up of a marketing/commercialization representative, a technical representative, a scientific representative, and a regulatory representative, etc. The team’s goal is to determine all the high-level requirements for the device. These could be user specific such as how the device will interface with the human, any size considerations. The development team first creates the user and market inputs (i.e answering the questions: Who will use this device? How will it be used? What is the indication for use? Where will the device be used? What features are key to the success of the device both therapeutically and in the market?, etc.). These can be technology specific such as how the device will work or what defines success in reaching the ultimate goal of the device function. These should include any inputs from the regulations and applicable standards as well and these also should include key marketing inputs to the device. The final input is a risk evaluation of the device. Risks should be reviewed and integrated into all parts of this process. Reference ISO 14971:2019 *Medical devices — Application of risk management to medical devices* for specific guidance to this process (ISO, 2019).

The next step is to take the user and market input level and dig down deeper into the engineering level requirements that will support the designers and engineers in developing the device. This takes each user and market input and makes it more specific (which is why often these are called specifications). An example of this translation of user need to engineering detail is: *The user requires the device to be small. The engineering specification (or requirement) defines small as having less than 5 mm* (Cho et al., 2020) *in volume.* In this way, the engineer has a metric to determine how to design the product. The user and market requirements and engineering specification (or requirements) are considered: *design input*.

The team then designs the product and defines the design of the product and builds the product. This is a simple step to explain in general, but often takes years or decades to design, iterate, and perform bench top testing to reach the goals set in the design inputs. Once a final design set is determined the design is frozen and -verification testing phase- can begin. The goal of the verification phase is to prove out of the technology for safety and effectivity and to ensure the device meets the requirements and standards identified in the earlier stages. As discussed earlier, it is prudent to initiate some of the testing during the design and development phase, but the verification and validation (V&V) testing shows that the device reliably (read with statistical significance) meets the requirements and regulations and that the device is safe and effective. The V&V test results are a fundamental part of the packet submitted to the FDA as evidence that the device is ready to be sold to and used by the general medical community. The development and testing process is guided by the ISO 13485 standard for medical devices (ISO: International Organization for Standardization) (ISO, 2016).

Verification testing challenges all aspects of the device from the perspective of electrical, software, body response/chemical, mechanical, etc. The engineering work in this phase requires a knowledge of statistics and verification and validation principles and test method verification. The team fully develops the test plan based on the requirements and standards and executes the testing required using the Engineering Specifications. The engineers need to be able to research applicable standards to find standard test methods, to write or modify test methods as needed (test methods that are new or modified off the standard will require test method validation and/or rationale as to why they needed to be modified), use good scientific principles to write test protocols, have the test executed (or execute the test themselves), and finally analyze the data and draw conclusions that will be found in various reports. Analysis of the data is typically required to be statistically significant or justified, so a working knowledge of statistics is needed. The quality and regulatory team supports this activity since the results need to be written up and provided for the FDA approval of the device.

Another aspect of testing the device includes clinical, field, or animal trials (see Section 11). These tests occur for various purposes. They can be to gain insight from the user group as part of the human factors engineering, the tests can be to gain insight into the effectiveness of the device, or to gain insights into the risks or to inform details in preparing the accompanying instructions that might be needed. From the perspective of human factors and usability, the testing is in the form of formative and summative tests with user groups where impressions and errors from the end user groups are collected. Effectivity is typically tested in animals and/or clinical trials. Good testing practices and test method development are required to ensure that the data is not skewed or lead to erroneous conclusions (either accidentally or purposefully). Statistical methods are again an important part as well as technical writing of protocols, test cases, and reports.

The final stage prior to submitting for FDA approval is *validation testing*. Validation testing is more typically clinical in nature looking at the device holistically and validating that the final device product meets the market/user requirements in its final use cases. To achieve this testing either clinical testing itself or reliable replacements for clinical tests. In the case of Class III medical devices much of the validation testing can only be done on the first in man devices that will require an investigational device exemption approval from the FDA prior to clinical testing and ultimate device approval for marketing. Critical aspects of the V&V testing are preclinical animal testing, both described in detail in the following sections.

## 11 Preclinical studies (*K. L. Vincent*)

**Figure 12:**
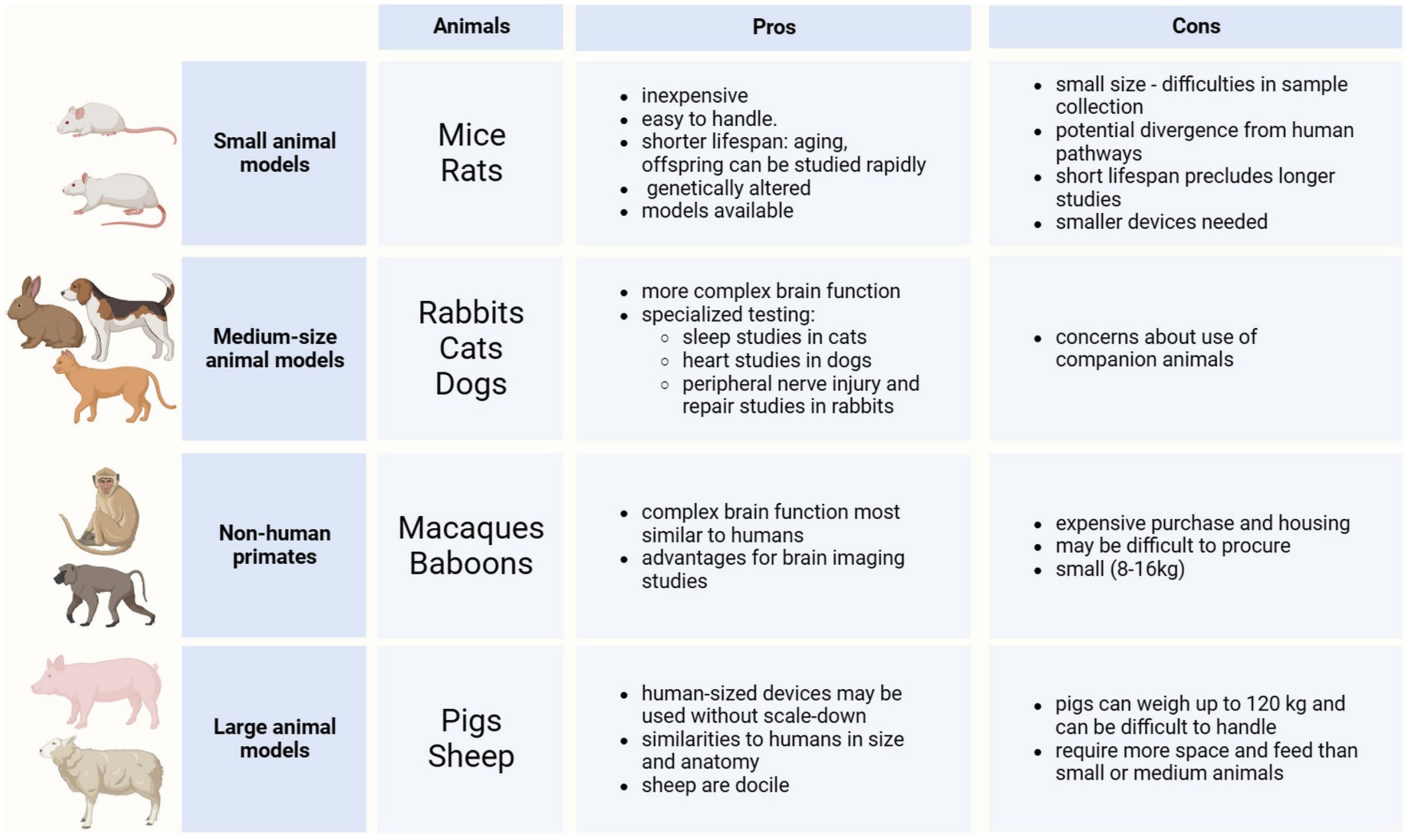
Animal models used in preclinical studies. Created with BioRender.com.

Preclinical models are necessary for many steps in the development of medical devices, from answering basic science questions to testing the design engineering and performance prior to use in humans (see Section 9). Preclinical testing with the use of animal models allows for hypothesis testing, mapping neural pathways and exploring the therapeutic potential of nerve stimulation (Swanson et al., 2012; Frasch et al., 2016; Gierthmuehlen and Plachta, 2016; Douven et al., 2020). Animal models also allow for invasive testing during the development process that is not feasible in humans, including adjustment of device size, fit and design, and for optimization of settings such as frequency, timing, duration, and location of treatment in preparation for clinical studies (Figure 12).

Animal models typically include mammals, from small species such as rodents, to larger, including sheep. When selecting an animal model it is important to consider the size and accessibility of the desired target (brain or periphery), and the similarity to humans in pathway, size, and function while being cognizant of the advantages and disadvantages of each. Additionally, bioethical considerations must be taken into account when working with live animals. Small mammals such as mice and rats are inexpensive to purchase, house, and feed. They are easy to manage and handle. They have a shorter lifespan so offspring and aging can be studied more rapidly (Vandamme, 2014). Additionally, there are many genetically altered mouse models available, including transgenic, knock-out, and knock-in mice models, to allow for the study of various mechanisms and pathways. However, the disadvantages include the small size, nerve structures and vessels for blood and sample collection as well as the potential divergence of pathways from those of humans. A shorter lifespan can preclude longer studies while the small size requires that smaller devices are used to fit.

Mid-size animals such as rabbits, cats, and dogs have more complex brain functions than rodents and may be used for specialized testing such as sleep studies for cats or studies on the heart for dogs (Pinnapureddy et al., 2015; Sánchez-León et al., 2018), whereas rabbits have been used extensively in peripheral nerve injury and repair studies (Kim et al., 2020; Koppaka et al., 2022; Sorkin et al., 2022). Non-human primates (NHP), with complex brain functions most similar to humans, offer advantages for brain imaging studies, however are costly and may be difficult to procure (Milham et al., 2020; Song et al., 2021; Hartig et al., 2023; Suminski et al., 2023). Additionally, most NHP are small, with rhesus macaques weighing 8–12 kg, and the largest research NHPs, baboons, weighing ~16 kg (Nakamura et al., 2021; Song et al., 2021). Larger animals such as pigs and sheep offer advantages in device development in that human-sized devices may be used without scale-down due to similarities in size and anatomy to that of humans. While sheep are docile and typically weigh less than 80 kg, pigs can weigh up to 120 kg and can be difficult to handle (Upton, 2008). They both require more space and feed, and thus higher daily care costs, than small mammals.

While working with companion animals has been thought to be more related to stress and burn-out in research lab workers, studies have shown that emotional bonding can occur with any species, that animal welfare is improved with strong human relationships, and that communication and institutional support programs are important for the well-being of those who work with research animals (LaFollette et al., 2019, 2020).

The FDA offers guidance documents for conducting preclinical and clinical studies for the development of medical devices. Previously for preclinical studies, this guidance was device type-specific, however, more recently (March, 2023), the FDA has provided general guidance for animal studies intended to evaluate medical devices (FDA, n.d.). While the guidance documents state that the suggestions are non-binding, it reflects the review process at the FDA and provides a standardized process for device developers to follow. For specific recommendations, the documents repeatedly advise submitting a pre-submission to seek personalized FDA feedback. The primary objective of animal studies is to provide assurance of safety in a biological setting and during use and performance of the device. However, the FDA recognizes that efficacy testing, proof of concept, and design development, among other objectives may also be achieved in animal testing. In brief, FDA guidance recommends: (1) identifying all risks, local and systemic, associated with the device procedure, use, and performance device and designing the study objectives to assess these risks, (2) using the most appropriate animal model based on previous studies in similar devices, or if not previously reported, based on consultation with a veterinarian based on anatomy and with FDA feedback, (3) with the least number of animals based on sample size calculation, (4) providing appropriate anesthesia and analgesia and monitoring before, during, and after the procedure for signs of adverse effects, with (5) post-mortem studies including necropsy and histology evaluation, and (6) performed under Good Laboratory Practices (GLP).

The following examples demonstrate how different animal models have been important for research into mechanisms and pathways and then utilized in the testing of VNS and devices. Implantable VNS devices have been available since 1997 for the treatment of epilepsy, 2005 for depression, and 2015 for obesity (Morris et al., 2013; Guiraud et al., 2016; Austelle et al., 2022), with scientific contributions from animal studies reported as early as 1952 (Zanchetti et al., 1952). Animal model research has continued to provide insight into VNS stimulation potential for the treatment of fibromialgia (Martins et al., 2021), headaches (Yuan and Silberstein, 2017), arthritis (Levine et al., 2014; Koopman et al., 2016; Zachs et al., 2019), inflammatory bowel disease (Bonaz et al., 2021; Fornaro et al., 2022), asthma (Tornero et al., 2022) and motor recovery after stroke (Hays et al., 2016; Capone et al., 2017; Morrison et al., 2021). Preclinical research with mice, rats, rabbits, cats, and dogs, as well as cadaver studies were utilized in mapping studies to determine the neural pathways and in nerve stimulation studies that preceded the development of the devices (Kaniusas et al., 2019; Wang et al., 2021). A study comparing vagal nerve size and histologic characteristics of mice, rats, dogs, pigs, NHPs, and humans suggested that a contributor to failed clinical studies using a vagal nerve stimulation device to treat heart failure was the difference in the size and characteristics of the animal models used to develop the device.

Therefore, a prudent path to device development could include the use of smaller animal models for exploration of neural pathways and nerve stimulation and for early prototypes of the device, with larger animals used for scale-up in the size and optimization of working parameters of the device prior to clinical studies. The pros and cons of each animal model must be considered when choosing a model for device testing in order to optimize the results and relevance for translation to humans. The ultimate purpose of the use of animals in preclinical studies is for the translation of treatments and diagnostics into human use so that new medical discoveries can be implemented into clinical care.

## 12 Neuromodulation and clinical applications (*B. Ma*)

As a last section, we will provide an overview of the critical aspects to consider when working with patients and implementing neuromodulation therapies.

Neuromodulation therapies have been in continuous development, and from the preclinical validation to the human intra-operative and post-operative processes, we continue to gain insights into their modes of function and their potential clinical therapeutic benefits (Sánchez-León et al., 2018; Gupta et al., 2020; Galli et al., 2020; Piper et al., 2022; Hays et al., 2023). As we have discussed, neuromodulation has widely changed the landscape of clinical treatment in various fields, including movement disorders, inflammatory disease, stroke, headache, pain, depression, and epilepsy. While traditional pharmacologic therapies often come with significant short- and long-term systemic side effects, neuromodulatory devices are well-tolerated and effective adjunctive treatments (Giordano et al., 2017; Won et al., 2020; Pavlov and Tracey, 2022). Targets may include a particular abnormal brain focus or a neural network and are flexible in their stimulation parameters and treatment cycles. Since a special issue would be needed just to discuss the various clinical applications of neurostimulation and their impacts on their respective fields, we will elaborate here on just one example of a disease state that has benefited greatly from the introduction of neuromodulatory therapies, namely drug-resistant epilepsy.

Between 30–40% of people with epilepsy are categorized as drug-resistant (Kwan and Brodie, 2000) and have incomplete responses to pharmacologic therapies (Brodie et al., 2012; Chen et al., 2018). Individuals who do not qualify for more definitive surgical treatments may benefit from neuromodulation (Nune et al., 2015; Salanova et al., 2015; Nair et al., 2020; Kostov et al., 2022). Epilepsy is increasingly viewed as a disease of abnormal neural networks, and disruption of these networks via neuromodulation can effectively decrease the frequency and severity of seizures. Three devices are currently approved for the treatment of drug-resistant epilepsy, including VNS, DBS, and responsive neurostimulation (RNS). Although each device functions differently, efficacy appears comparable, with more than half of individuals having a greater than 50 % reduction in seizure frequency (Salanova et al., 2015; Nair et al., 2020; Kostov et al., 2022). Seizures are characterized by abnormal synchronized electrical activity, and desynchronization and modulation of these epileptogenic neural networks, either by interrupting key propagation points or focally disrupting seizure onset zones are potential mechanisms of neuromodulation (Piper et al., 2022). There is growing evidence that these devices not only acutely disrupt epileptogenic activity, but also allow for long-term stabilization of underlying networks and alteration of connectivity patterns (Khambhati et al., 2021; Piper et al., 2022), helping to explain the trend that efficacy of these devices improve with time (Salanova et al., 2015; Nair et al., 2020; Kostov et al., 2022).

### 12.1 Open-loop systems

Open-loop systems in neuromodulation provide continuous or scheduled intermittent therapy that is delivered with no real-time biological feedback. The VNS and DBS provide scheduled intermittent therapy to standard implantation targets (Hays, 2016; Yuan and Silberstein, 2016; Peltola et al., 2023) and stimulation parameters including output current, frequency, pulse width, and on/off times can be titrated and adjusted over time. These devices indirectly stimulate the neural networks involved in seizure generation and propagation, causing diffuse neuromodulatory effects.

VNS provides purely extracranial stimulation, targeting the afferent innervations of the vagus nerve. The generator is placed through a small incision in the chest and the electrode is wrapped around the left vagus nerve. This device is generally well-tolerated, although common side effects include coughing, hoarseness, and dysphonia with stimulation (Ramsay et al., 1994). Stimulation of the vagus nerve leads to widespread downstream targets for neuromodulation, affecting central noradrenergic and serotonergic pathways through modulation of the nucleus tractus solitarius, locus coeruleus, and raphe nuclei (Krahl and Clark, 2012), as well as altering thalamic and other limbic networks (Ben-Menachem, 2002). Battery life depends on the output current and stimulation frequency, but on average lasts between 5–7 years. Current technologies are focusing on the development of wireless and battery-less stimulators (Habibagahi et al., 2022).

DBS provides intracranial stimulation via depth electrodes that target various brain regions involved in the particular disease process in question, such as the subthalamic nuclei or globus pallidus interna for Parkinson’s disease and the thalamus for epilepsy. The DBS generator is implanted in the chest. In epilepsy, the thalamus serves as a common relay station and propagation point in many cortico-subcortical epileptogenic networks. High-frequency stimulation may inhibit seizure propagation as well as disrupt the epileptogenic network (Piper et al., 2022). The anterior nucleus of the thalamus, a part of the circuit of Papez, is an integral node in many neural networks, connecting the mesial and anterior frontal and temporal regions. Targeting this nucleus can have positive benefits in epileptogenic networks involving the limbic system (Salanova et al., 2015). The most frequently reported adverse events with implantation of the anterior nucleus of the thalamus include depression and memory impairment (Salanova et al., 2015; Peltola et al., 2023). Increasing evidence also suggests that stimulation of the centromedian nucleus of the thalamus may be effective in individuals with generalized epilepsies (Velasco et al., 2021; Vetkas et al., 2022).

### 12.2 Closed-loop systems

Closed-loop neuromodulation refers to the automatized delivery of electrical therapy in real-time in response to a pre-defined biomarker in the patient. This type of neuromodulation has been referred to as responsive neurostimulation (David et al., 2019; Nair et al., 2020; Assenza et al., 2021; Sisterson et al., 2022).

The RNS system provides electrical stimulation in response to specific patterns of abnormal neural activity, now widely used as a treatment for drug-resistant epilepsy (Morrell, 2011; ISO, 2016). The generator is placed directly over the brain via a full thickness craniectomy and the electrodes are targeted either to the specific epileptogenic zone identified in a patient or to standardized sites like the thalamus that may be involved in the epileptogenic network (Elder et al., 2019; Sisterson et al., 2022). Detection of interictal and ictal epileptiform activity in turn triggers stimulation of the underlying cerebral tissue in attempts to suppress or desynchronize the epileptogenic network (Piper et al., 2022). RNS implantation is highly individualized and requires close collaboration between the neurosurgeon and neurologist. Benefits include the output of long-term electrocorticography, which allows for tailored therapy specific to a person’s unique seizure pattern. Responsive neurostimulation is now also under investigation for uses in neuropsychiatric conditions like major depression (Scangos et al., 2021).

### 12.3 Patient experience

Neuromodulation in epilepsy has significantly increased the therapeutic options available in those with limited remaining pharmacologic options, showing demonstrable improvements in quality of life and decreased rates of sudden unexpected death in epilepsy persons (SUDEP) (Nair et al., 2020; Kostov et al., 2022), one of the most important markers in epilepsy. Disadvantages include that the therapeutic benefits are not immediate and often require months to years to achieve maximal benefit (Salanova et al., 2015; Nair et al., 2020; Kostov et al., 2022). Unlike medications, however, which often cause significant side effects of sedation and cognitive slowing, stimulation from these devices is generally asymptomatic or very well-tolerated (Ben-Menachem, 2002; Nair et al., 2020; Peltola et al., 2023). Neuromodulation also provides an option for neurocognitively intact individuals when resective surgery of eloquent areas may impact cognition or memory (Chen et al., 2018).

### 12.4 Future directions in clinical applications

One of the key questions in neuromodulation is understanding which patients may benefit the most from neurostimulation and determining optimal neuromodulatory targets. Neuromodulation therapies are being expanded to a broad cohort of applications, including but not limited to spinal cord stimulation for pain relief, carotid body stimulation for hypertension, pelvic floor nerves stimulation for urinary incontinency, hypoglossal nerve for obstructive sleep apnea, and VNS for inflammatory pathologies (Pavlov et al., 2020; Pavlov and Tracey, 2022; INS, n.d.). Some therapies have standard targets across all individuals, making implantation techniques and stimulation parameter titration straightforward, while others, such as with RNS therapy in epilepsy, involve more personalized evaluations and individualized therapy regimens. Ongoing research is helping us better employ pre-implantation data and identify biomarkers to guide clinical decisions in neurostimulation techniques.

## 13 Conclusion

One would expect treatment options to evolve in line with the technological, scientific, and medical revolution we are currently experiencing. In this sense, the arrival of Bioelectronic Medicine has ushered in the era of personalized and organ-targeted therapies that could overcome the main pitfall of drug therapies. Although drug therapies are being optimized to treat specific targets, their systemic delivery may still be associated with off-target adverse effects. Advances in Bioelectronic Medicine have the potential to eliminate off-target effects and adapt to individual needs at a specific time, enabling accurate and personalized medicine. Our view is however that pharmacological and bioelectronic treatments could be combined to maximize treatment effects while minimizing adverse and off-target effects.

Bioelectronic Medicine is cross-disciplinary in nature. Its advent therefore requires the joint effort of a multidisciplinary team not only to unravel the mechanisms of the disease, but also to optimize technologies that meet specific necessities and the data management to inform closed-loop and AI-driven systems to model more accurate and personalized treatments. The remaining challenges are not the least, they include a precise knowledge of all the elements contributing to synaptic transmission, ranging from ion channels to the integration of signals generated within circuits, including neuroglia in the broadest sense of the term and the elements involved in neuromodulation, as well as the impact of changes in the nervous system to the whole organism. Significant progress needs to be made on a number of technical aspects of neural interfaces and data processing. These aspects, together with regulatory implementation to secure patient safety, must be integrated to master neuromodulation as a therapeutic tool.

To finally conclude, we would like to express that this review has been a collaborative effort to encourage young readers, non-experts and experts to start or continue their efforts in the field of Bioelectronic Medicine, which holds the promise of rewriting the narrative of healthcare and presenting it with unprecedented possibilities in a new era of therapeutic alternatives.

## Author contributions

MAG-G: Conceptualization, Writing – original draft, Writing – review & editing. SC: Writing – review & editing. RL: Writing – review & editing. ST: Writing – review & editing. MP: Writing – review & editing. NCS: Writing – review & editing. AV: Writing – review & editing. M-ÈT: Writing – review & editing. CA: Writing – review & editing. AH-R: Writing – review & editing. ME: Writing – review & editing. JC: Writing – review & editing. KV: Writing – review & editing. BM: Writing – review & editing.

## Footnotes

^1^Most of the electrophysiological experiments mentioned in this section used *Xenopus laevis* oocytes as the expression system for the cloned ion channels. Of note, Ricardo Miledi developed the use of this channel expression system at the beginning of the 1980s (Barnard et al., 1982).

## Glossary

ACh: Acetylcholine
ACTH: Adrenocorticotrophic hormone
AD: Alzheimer’s disease
AI: Artificial intelligence
ALS: Amyotrophic lateral sclerosis
BBB: Blood brain barrier
BDNF: Brain-derived neurotrophic factor
BK: Ca2+ and voltage-activated channels
Cav: Voltage-dependent Ca2+ channels
CMS: Centers for Medicare and Medicaid Services
CNS: Central nervous system
CRH: Cortisol releasing hormone
CSC: Charge storage capacity
CSCc: Cathodal charge storage capacity
CRCNS: Collaborative Research in Computational Neuroscience
CV: Cyclic voltammetry
DBS: Deep brain stimulation
DMN: Dorsal motor nucleus
e0: Electronic charges
ECoG: Electrocorticogram arrays
EIS: Potentiostatic electrochemical impedance spectroscopy
Em: Membrane potential
Emc: Electrochemical potential excursion
Eoc: Open-circuit voltage
Epi: Epinephrine
FDA: Food Drug Administration
FTD: Frontotemporal dementia
GC: Glucocorticoids
GL: Leakage conductance
GLP: Good Laboratory Practices
GPa: Gigapascal
HER: Electronic health records
HH: Hodgkin and Huxley
IBD: Inflammatory bowel disease
IL: Interleukin
IN: Negative inward
INS: International Neuromodulation Society
IoT: Internet of Things
ISO: International Organization for Standardization
KCNH: Voltage-gated potassium channels
Kv: Voltage-dependent K+ channel
LPS: Lipopolysaccharide
Max Qinj: Maximum charge injection capacity
MPa: Megapascal
N: Number of channels
Nav: Voltage-dependent Na+ channel
NE: Norepinephrine
NHP: Non-human primates
NIH: National Institute of Health
NSF: National Science Foundation
NTS: Nucleus tractus solitary
OCP: Open circuit potential
OPC: Oligodendrocyte precursor cells
P: Permeability coefficient
Parylene C: Poly(chloro-p-xylylene)
PBS: Phosphate-buffered saline
PD: Pore domain
PD: Parkinson’s disease
PDMS: Polydimethylsiloxane
PEDOT: Poly(3,4-845 ethylenedioxythiophene)
PI: Polyimide
PNS: Peripheral nervous system
PVN: Paraventricular nucleus
Q: Total gating charge
qBBr: Fluorescent positively charged bimane derivative
RNS: Responsive neurostimulation
S1-6: Six transmembrane segments
SMP: Shape memory polymer
SUDEP: Sudden unexpected death in epilepsy persons
Ti-N: Titanium nitride
TMS: Transcranial magnetic stimulation
TNF: Tumor necrosis factor
TTX: Tetrodotoxin
tVNS: Transcutaneous auricular vagus nerve stimulation
V & V: Verification and validation
VNS: Vagus nerve stimulation
VSD: Voltage sensor domain
VT: Voltage transient
α7nAchR: Nicotinic acetylcholine receptor subunit α7
